# Super-Sensitive Chemiluminescent Probe for the Detection of Caspase‑3 Activity

**DOI:** 10.1021/acs.bioconjchem.5c00151

**Published:** 2025-05-08

**Authors:** Rozan Tannous, Chi Zhang, Doron Shabat

**Affiliations:** † School of Chemistry, Raymond and Beverly Sackler Faculty of Exact Sciences, 26745 Tel-Aviv University, Tel Aviv 69978 Israel; ‡ School of Chemistry and Chemical Engineering, 12443 Huazhong University of Science and Technology, Luoyu Road 1037, Wuhan 430074, China

## Abstract

Caspase-3 is a pivotal enzyme in the apoptosis pathway that is responsible for executing programmed cell death through the cleavage of key cellular proteins. Existing fluorescence-based probes for caspase-3 detection suffer from limitations such as background noise from tissue autofluorescence and light scattering, reducing their sensitivity and real-time imaging capabilities. To overcome these limitations, we developed a chemiluminescent probe, Ac-DEVD-CL, that enables the highly sensitive and selective detection of caspase-3 activity. Upon caspase-3-mediated cleavage, the probe undergoes a self-immolative reaction that triggers a chemiluminescent signal, allowing real-time monitoring of the enzymatic activity. Probe Ac-DEVD-CL demonstrated an exceptionally high turn-on response, with a 5000-fold increase in the chemiluminescent signal upon enzymatic activation. The probe exhibited notable specificity for caspase-3, with minimal cross-reactivity toward other biologically relevant proteases and tumor-associated enzymes. Additionally, inhibition studies using the caspase-3 inhibitor confirmed that the probe’s activation is exclusively mediated by caspase-3. A direct comparison with the commercially available fluorescent probe revealed that probe Ac-DEVD-CL offers significantly improved sensitivity, achieving a signal-to-noise ratio 380-fold higher and a limit of detection 100-fold lower. These results establish probe Ac-DEVD-CL as a highly effective tool for detecting caspase-3 activity with superior precision. Finally, we validated the probe’s utility in imaging apoptosis in live cells. In 4T1 breast cancer cells treated with cisplatin, Ac-DEVD-CL generated a strong chemiluminescent signal, with a three-order-of-magnitude enhancement compared to untreated cells. Overall, the probe Ac-DEVD-CL demonstrates a significant improvement in detection sensitivity, providing a powerful and versatile chemiluminescent probe for real-time imaging of caspase-3 activity. Its exceptional sensitivity and selectivity could make it a valuable tool for cancer research, drug discovery, and therapeutic monitoring.

## Graphical abstract

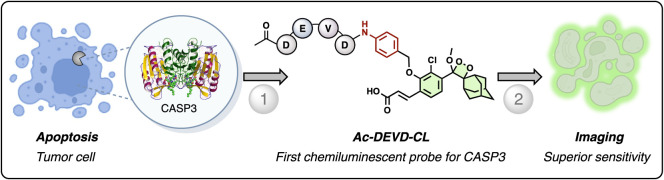

## Introduction

Caspase-3 is a key executioner protease in the apoptotic cascade, playing a crucial role in programmed cell death through the cleavage of structural and regulatory proteins.^1^ Due to its central function, the ability to accurately detect and quantify caspase-3 activity is essential for studying apoptosis in various biological contexts, including cancer, neurodegenerative disorders, and drug-screening applications.^2–6^

The apoptotic process is heavily regulated by caspases, a family of cysteine-dependent proteases that mediate the signaling cascades leading to cell death.^7^ Among them, caspase-3 is an important key executor in cell apoptosis and is the most studied and characterized member of this family.^8^ Several studies have implicated caspase-3 as an important effector enzyme in promoting therapy-induced cell death.^9^ Moreover, dysregulated caspase-3 activity has been linked to resistance to drug-induced apoptosis in tumor cells.^3,10^ Therefore, probes that enable real-time monitoring of caspase-3 activity are invaluable for tracking apoptosis and predicting responses to anticancer therapies.

Several fluorescence-based probes have been developed to visualize apoptosis by detecting caspase-3 activity.^11–32^ However, these probes require external light sources, resulting in increased background noise from tissue autofluorescence and light scattering, which limits sensitivity and real-time detection capabilities.^33^ The limitations of the fluorescence probes prompted us to explore an alternative optical approach for probe design. Chemiluminescence has emerged as a powerful and versatile tool for diagnostic and imaging applications.^34^ Unlike fluorescence, chemiluminescence generates light via a chemical reaction, eliminating the need for an external light source. This intrinsic property reduces background interference and consequently leads to enhanced signal-to-noise ratios, making chemiluminescent probes among the most sensitive tools available for detecting enzymatic activities.^35,36^

Recent advancements in chemiluminescent probe technology achieved by our group have been driven by the discovery of *ortho*-substituted phenoxy-dioxetanes.^37^ Specifically, incorporating an acrylate substituent at the *ortho* position of a phenoxy-adamantyl-1,2-dioxetane luminophore prevents water-mediated quenching of the excited intermediate, resulting in a remarkable 3000-fold increase in light emission intensity. These novel chemiluminescent luminophores have been extensively utilized to develop highly sensitive turn-on probes for detecting various bioanalytes and enzymatic activities.^38–55^ Here, we present the design, synthesis, and evaluation of the first chemiluminescent probe (**Ac-DEVD-CL**) for the real-time detection of caspase-3 enzymatic activity. This probe leverages the advantages of chemiluminescence to achieve highly sensitive and selective detection of apoptosis, therefore providing a powerful tool for monitoring drug-induced cell death.

## Results and Discussion

Caspase-3 (CASP3) is a cysteine-aspartic acid protease known to specifically hydrolyze the C-terminal amide bond of the peptide substrate Asp–Glu–Val–Asp (DEVD).^56^ Therefore, we designed a probe in which the caspase-3-specific substrate (Ac-DEVD) is coupled via a self-immolative linker to a phenoxy-1,2-dioxetane luminophore bearing an acrylic acid substituent at the *ortho* position. Caspase-3 can catalyze the cleavage of the Ac-DEVD substrate, followed by 1,6-elimination of azaquinone methide, which leads to the release of the phenoxy-1,2-dioxetane. The latter then undergoes a chemiexcitation disassembly that results in the release of an excited benzoate species, which rapidly decays through the emission of a green photon (Figure 1A).

**Figure 1:**
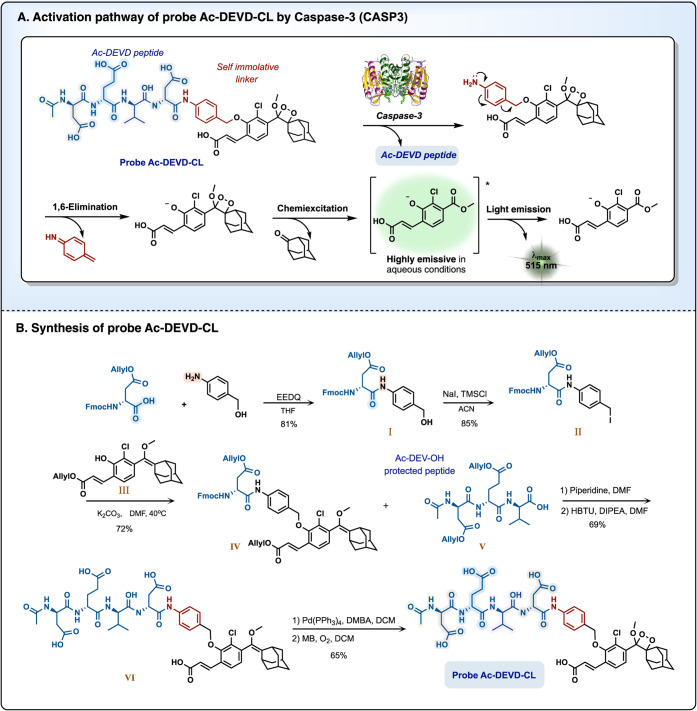
Probe **Ac-DEVD-CL** (A) chemiluminescent activation pathway upon reaction with caspase-3. (B) Synthetic route used to prepare probe **Ac-DEVD-CL**.

The synthesis of probe **Ac-DEVD-CL** was achieved according to the synthetic route described in Figure 1B. l-amino acids were used to synthesize the corresponding caspase-3 substrate. First, Fmoc-Asp­(OAll)–OH was coupled with *para-*aminobenzyl alcohol to form amide **I**. The latter was then treated with sodium iodide and trimethylsilyl chloride to produce benzyl iodide **II**. Nucleophilic substitution of benzyl iodide **II** by a previously synthesized phenol enolether **III** afforded enolether **IV**. Next, the Fmoc-protecting group of compound **IV** was removed by piperidine, yielding a free amine intermediate. This amine was then coupled with the allyl-protected tripeptide Ac-Asp­(OAll)-Glu­(OAll)-Val-OH (Ac-DEV, **V**), synthesized via solid-phase peptide synthesis (SPPS), to generate amide **VI**. Finally, removal of the four allyl ester-protecting groups using a Pd(0) complex, followed by oxidation with singlet oxygen, yielded probe **Ac-DEVD-CL**.

With the probe **Ac-DEVD-CL** in hand, we initially sought to evaluate its turn-on chemiluminescent response upon reaction with the active human recombinant caspase-3 (Figure 2A). The chemiluminescent kinetic profile of the probe **Ac-DEVD-CL** was measured in the presence and absence of caspase-3 under physiological conditions (HEPES buffer, pH 7.5). As shown in Figure 2B, the probe **Ac-DEVD-CL** exhibits a typical chemiluminescence kinetic profile upon incubation with caspase-3, with an initial light-emission increase to a maximum, followed by decay of the signal over more than 5 h. In contrast, the probe demonstrated almost no light emission in the absence of caspase-3. Remarkably, the total light emission measured by probe **Ac-DEVD-CL** in the presence of caspase-3 was 5491-fold greater than that observed in the absence of the enzyme (Figure 2C).

**Figure 2:**
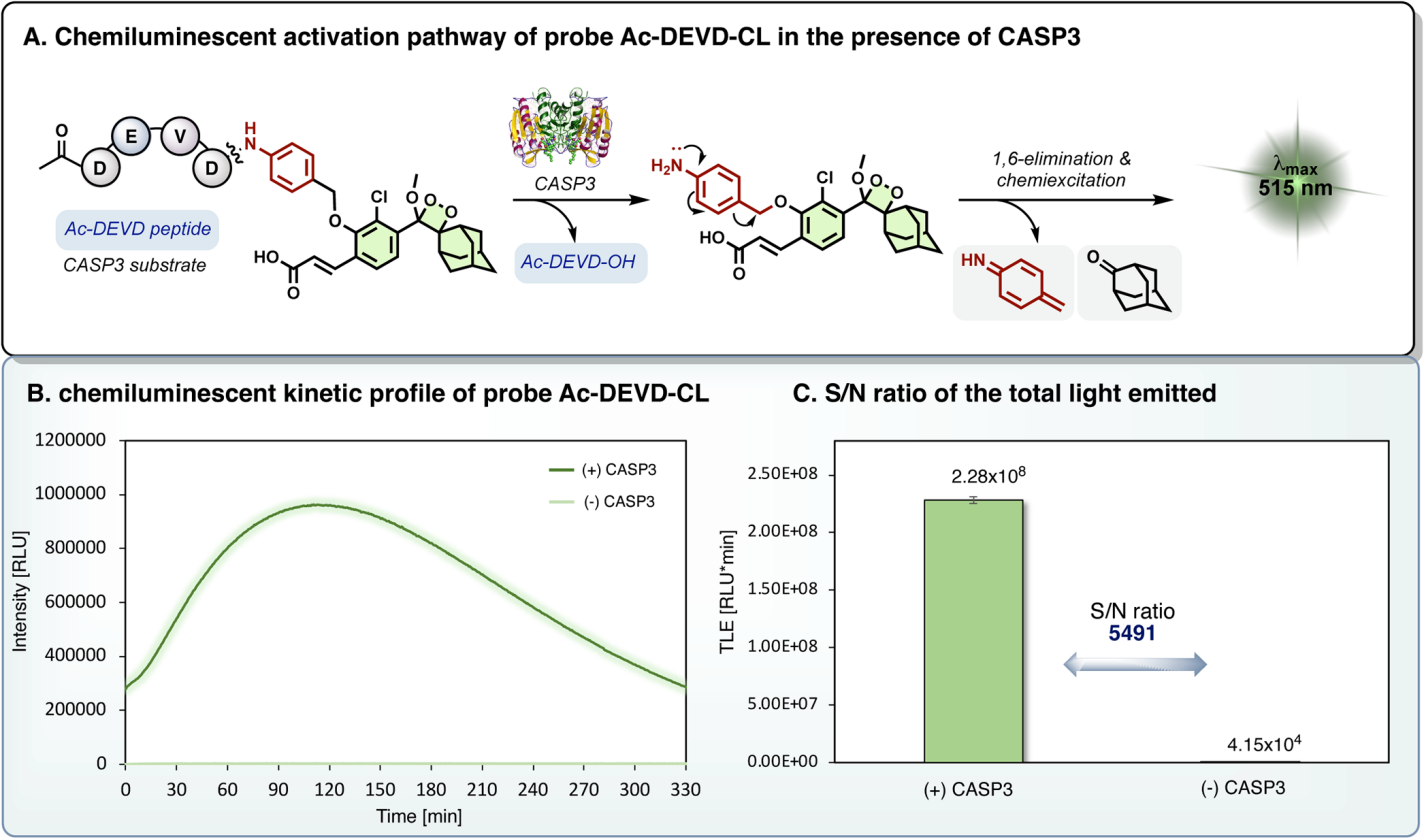
(A) Chemiluminescent activation pathway of the probe **Ac-DEVD-CL**. (B) Chemiluminescent kinetic profile and (C) total light emission of the probe **Ac-DEVD-CL** (10 μM), in HEPES buffer (pH 7.5, 1% DMSO, 1 mM DTT), at 37 °C in the presence or absence of recombinant human caspase-3 (1.66 μg/mL).

To evaluate the specificity of the probe **Ac-DEVD-CL** for its target enzyme (caspase-3), we assessed its chemiluminescence response toward other biologically relevant enzymes, including several known proteases (cathepsin B, trypsin, and aminopeptidase M) and common tumor-associated glycosidases (β-galactosidase, β-glucuronidase, and β-glucosidase). As shown in Figure 3A, the probe **Ac-DEVD-CL** exhibited high specificity as a substrate for detecting caspase-3 activity, resulting in an intense light emission signal with a signal-to-noise (S/N) ratio of 901-fold. This S/N value is approximately 3 orders of magnitude greater than the values observed with all other tested enzymes (S/N values of 1.0–4.4). The exceptional selectivity observed for the probe **Ac-DEVD-CL** emphasizes its suitability for detecting caspase-3 activity in complex biological environments.

**Figure 3:**
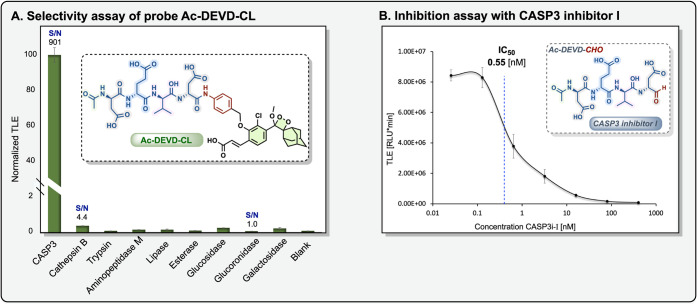
(A) Substrate specificity evaluation of the probe **Ac-DEVD-CL** (10 μM) in the presence of nine commercially available enzymes (caspase-3 (0.5 μg/mL), cathepsin B (1.4 U/mL), trypsin (2 U/mL), aminopeptidase M (2 U/mL), lipase (2 U/mL), esterase (2 U/mL), β-glucosidase (10 U/mL), β-glucuronidase (2 U/mL), and β-galactosidase (2 U/mL)). (B) Total light emitted (TLE) after 4 h of incubation with the probe **Ac-DEVD-CL** (10 μM) and caspase-3 (0.15 μg/mL), preincubated for 30 min with varying concentrations of caspase-3 inhibitor (CASP3i, 400–0.0256 nM) in HEPES buffer (pH 7.5, 1% DMSO, 1 mM DTT), at 37 °C.

To further confirm the specificity of the probe, we examined whether the probe activation could be inhibited by Ac-DEVD-CHO (CASP3i), a known caspase-3 inhibitor. Therefore, we coincubated the enzyme with increasing concentrations of CASP3i for 30 min before the addition of the probe **Ac-DEVD-CL**. As presented in Figure 3B, the chemiluminescent signal was inhibited with the increase in inhibitor concentration by a sigmoid inhibition behavior. At the highest concentration of 400 nM, the inhibitor reduced the chemiluminescent signal by more than 98%, confirming that the activation of the probe **Ac-DEVD-CL** is exclusively mediated by caspase-3.

As mentioned above, chemiluminescent probes have an inherent advantage over fluorescent probes. The request for an external light excitation source in fluorescence generates a substantial noise signal, leading to a compromised sensitivity. On the contrary, in chemiluminescence, the excited state of the emitter is formed through the disassembly of energetic chemical bonds. When the molecule has high chemical stability, this mode of excitation practically produces an extremely low noise signal. To demonstrate the advantage of the chemiluminescent probe, we compared the detection sensitivity of probe **Ac-DEVD-CL** with that of the commercially available fluorescent probe **Ac-DEVD-AMC**. The latter is an analogous fluorescent probe composed of aminomethyl-coumarin (AMC) masked with the same peptidyl substrate used in our chemiluminescence probe (Figure 4A).^57^ We next measured the S/N ratios of both probes upon incubation in the presence and absence of active caspase-3 under identical conditions. As shown in Figure 4B, both probes exhibited a significant turn-on response upon incubation with caspase-3 with a stable background signal. However, the S/N ratio observed for the probe **Ac-DEVD-CL** was 389-fold higher than that obtained for the probe **Ac-DEVD-AMC**.

**Figure 4:**
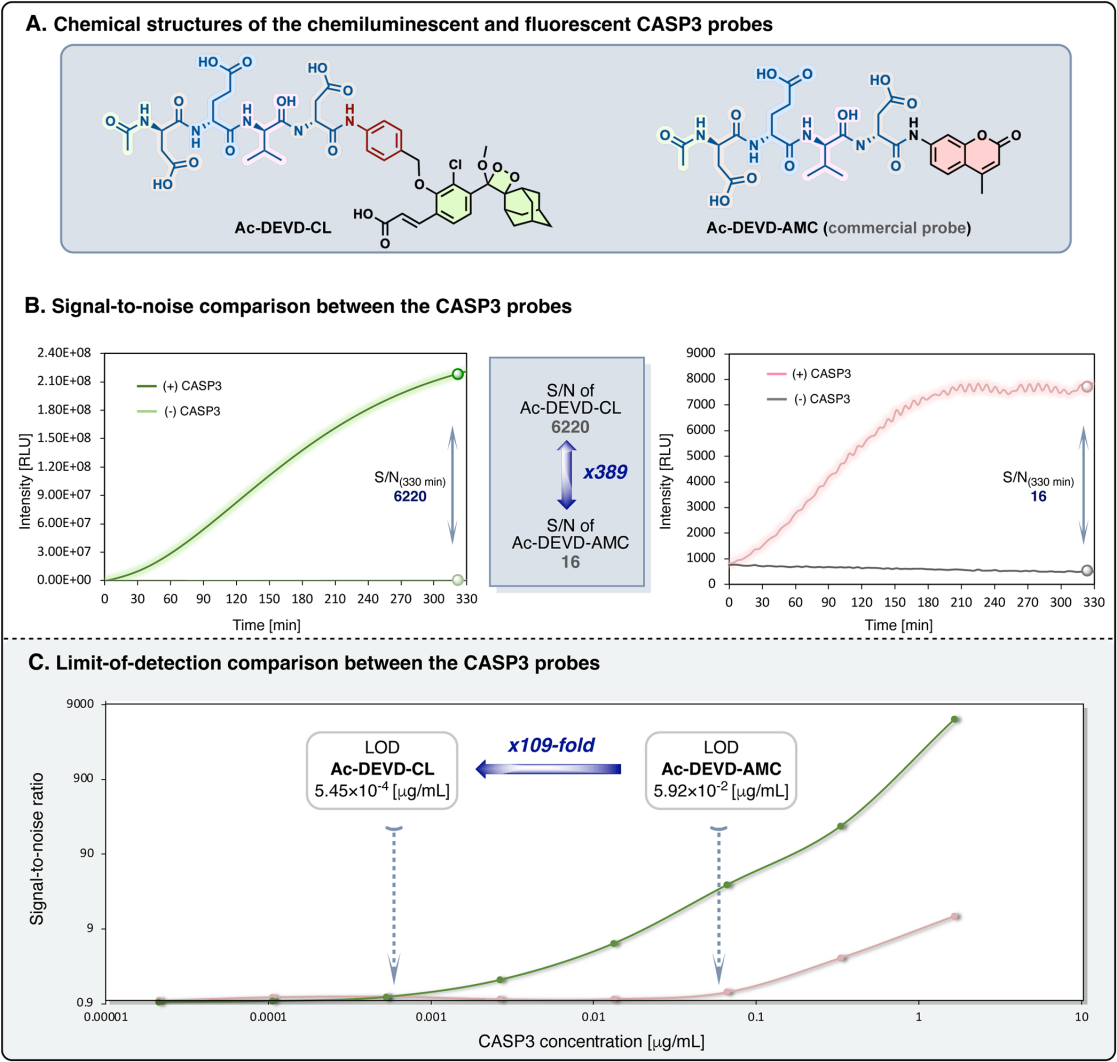
Comparison between the sensitivity of the chemiluminescent probe **Ac-DEVD-CL** and the commercially available fluorescent probe **Ac-DEVD-AMC**. (A) Chemical structure, (B) kinetic profile, and (C) limit-of-detection (LOD) of the probes **Ac-DEVD-CL** (10 μM) and **Ac-DEVD-AMC** (50 μM) in the presence or absence of various concentrations of recombinant human caspase-3 (1.66–2.12 × 10^–5^ μg/mL), in HEPES buffer (pH 7.5, 1% DMSO, 1 mM DTT), at 37 °C.

The probe **Ac-DEVD-CL** displayed a faster response with an increased S/N ratio compared to the corresponding fluorescence analog, enabling rapid and sensitive detection of caspase-3 activity. To further validate the improved detection sensitivity of the probe **Ac-DEVD-CL** over that of **Ac-DEVD-AMC**, we then conducted a comparative evaluation of the LOD (limit of detection) values by incubating both probes with varying concentrations of recombinant CASP3. Remarkably, the probe **Ac-DEVD-CL** exhibited an LOD value of 5.45 × 10^–4^ μg·mL^–1^, which was 109-fold more sensitive than the LOD value obtained with probe **Ac-DEVD-AMC** (Figure 4C).

The high specificity and sensitivity exhibited by the probe **Ac-DEVD-CL** toward the detection of caspase-3 activity prompted us to evaluate its ability to detect caspase-3 activity in drug-induced cell apoptosis. As mentioned above, caspase-3 serves as a key biomarker for apoptosis due to its central role in executing programmed cell death, making it an important marker for assessing the effectiveness of apoptosis-inducing treatments such as chemotherapy or targeted cancer therapies.^58^ Given the superb sensitivity and fast turn-on response exhibited by the probe **Ac-DEVD-CL** toward caspase-3 detection, we next investigated its capability to image apoptosis in mouse breast cancer cells 4T1.

Tumoral 4T1 cells were treated with cisplatin (Cisp), a widely used chemotherapeutic agent, to induce apoptosis and activate CASP3.^59–61^ The probe **Ac-DEVD-CL** was added to the cells, and its chemiluminescent light emission was recorded (Figure 5A). Untreated 4T1 cells exhibited no detectable chemiluminescent signal, whereas cisplatin-treated cells generated a strong signal with an intensity level 3 orders of magnitude higher than that of untreated cells (Figure 5B). This observation is consistent with the fact that caspase-3 is activated during apoptosis. To confirm that the detected signal was specifically attributed to caspase-3 activity among other apoptotic enzymes, 4T1 cells were cotreated with Cisp and a caspase-3 inhibitor. As expected, the signal observed for the inhibitor-treated cells was identical to that of the untreated cells, indicating that the inhibition of caspase-3 activity by the inhibitor prevented probe hydrolysis, thus validating the specificity of the signal for caspase-3 activity.

**Figure 5:**
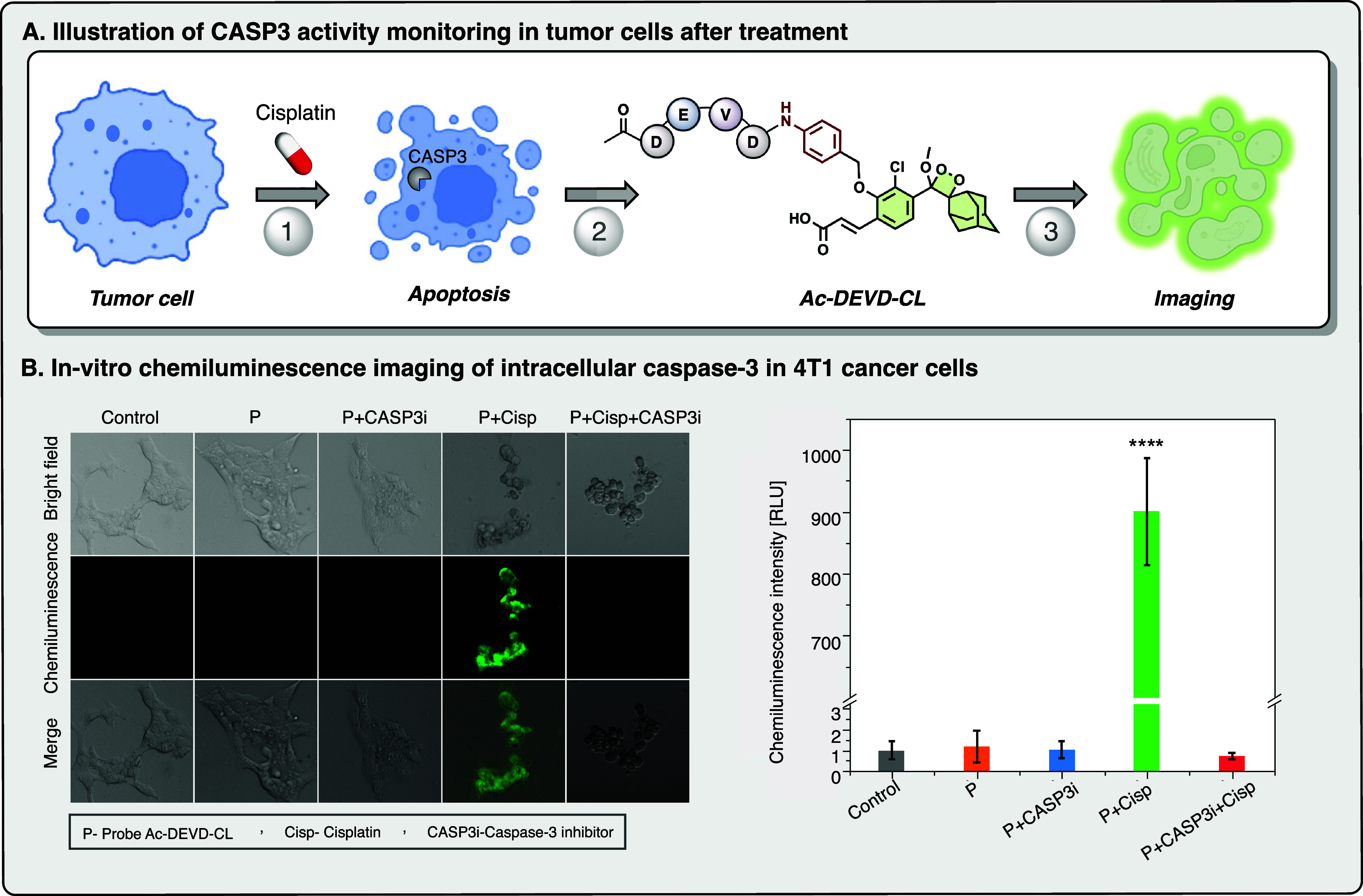
(A) Schematic representation of the caspase-3 activation pathway and its detection using the probe Ac-DEVD-CL during drug-induced apoptosis. (B) In vitro chemiluminescence imaging of intracellular caspase-3 in 4T1 cancer cells. (Left) Chemiluminescence images and (right) mean chemiluminescence intensity enhancement of 4T1 cells pretreated with cisplatin (Cisp, 300 μM), caspase-3 inhibitor (CASP3i, 100 μM) or both for 3 h before incubation with the probe **Ac-DEVD-CL** (10 μM) for 20 min (0.1% of DMSO). P+Cisp versus other groups: *p* < 0.0001. Statistical significance was calculated via one-way ANOVA with a Tukey post hoc test.

Morphological analysis confirms the minimal toxicity of the probe, as probe-treated cells exhibited a normal morphology. In contrast, significant apoptotic changes, including cell shrinkage, membrane blebbing, and eventual lysis, occurred only upon cisplatin treatment. Co-treatment with a caspase-3 inhibitor attenuated these effects, highlighting caspase-3′s role in cisplatin-induced apoptosis.

These findings confirm that the probe **Ac-DEVD-CL** specifically detects caspase-3 activity and can effectively image apoptosis in tumor cells. The remarkable 3 orders of magnitude signal enhancement in cisplatin-treated cells compared to untreated controls, combined with the complete signal suppression when using a caspase-3 inhibitor, demonstrates both the exceptional sensitivity and specificity of our probe for monitoring apoptotic processes in living cells.

In this study, we developed a new small-molecule chemiluminescent probe for the real-time detection and imaging of caspase-3 activity during apoptosis. Our results demonstrate that the probe **Ac-DEVD-CL** exhibits an exceptionally sensitive turn-on response upon enzymatic activation, significantly outperforming classic fluorescent probes like **Ac-DEVD-AMC**. Notably, the probe **Ac-DEVD-CL** achieved a limit of detection 109-fold lower than the commercially available probe **Ac-DEVD-AMC**, positioning it as a powerful tool for diagnostic applications in physiological conditions.

A major advantage of the probe **Ac-DEVD-CL** is its superior specificity for caspase-3, with minimal cross-reactivity with other proteases and tumor-associated enzymes. Inhibition studies using Ac-DEVD-CHO (CASP3i) further supported this specificity, showing a dose-dependent decrease in chemiluminescence, which confirmed that the signal originated specifically from caspase-3 activity. The ability to selectively and sensitively monitor apoptotic processes is crucial for both assessing disease progression and evaluating the effectiveness of therapeutic interventions. This is especially important in cancer treatment, where caspase-3 activation serves as a key marker for the apoptotic response to anticancer therapies, making reliable detection methods essential for evaluating treatment outcomes.

Cellular studies in 4T1 breast cancer cells demonstrated the probe’s practical utility in monitoring drug-induced apoptosis. A significant three-order-of-magnitude signal enhancement observed in cisplatin-treated cells relative to untreated controls validated the probe’s sensitivity in detecting caspase-3 activity in tumor cells undergoing apoptosis. This exceptional signal-to-noise ratio emphasizes the power of probe **Ac-DEVD-CL** for real-time, precise quantification of caspase-3 activity in tumor cells upon treatment. The complete signal suppression observed with caspase-3 inhibitor cotreatment further confirms the probe’s specificity in complex cellular environments. We anticipate that our findings on probe **Ac-DEVD-CL** will provide the scientific community with an efficient and highly sensitive tool for monitoring therapeutic responses based on caspase-3 activity. This advancement could enable real-time detection with unprecedented sensitivity, offering valuable insights into treatment efficacy and disease progression.

## Conclusions

In summary, we developed the first chemiluminescent probe for the selective detection of caspase-3 activity. By leveraging chemiluminescence, the probe overcomes limitations associated with fluorescence-based methods, achieving an exceptional signal-to-noise ratio and detection sensitivity. Probe **Ac-DEVD-CL** demonstrated remarkable specificity for caspase-3, with minimal cross-reactivity, and successfully imaged apoptosis in live tumor cells with high precision. Its ability to provide real-time, background-free detection of caspase-3 activity highlights its potential as a valuable tool for cancer research, drug discovery, and therapeutic monitoring.

## Supplementary Material



## Notes

Synthetic procedures and characterization of all compounds, including ^1^H-NMR, ^13^C-NMR, HPLC, and MS; experimental protocols; Figures S1–S8 (PDF)
