# Supplementary material for: Super-Sensitive Chemiluminescent Probe for the Detection of Caspase‑3 Activity
Source: Bioconjug Chem. 2025 May 8;36(5):1113–20. doi: 10.1021/acs.bioconjchem.5c00151 (PMC12100654; doi:10.1021/acs.bioconjchem.5c00151)
Supplement: Supplementary file 1 [file bc5c00151_si_001.pdf]

Supporting Information

Super Sensitive Chemiluminescent Probe for Detection of Caspase-3 Activity

Rozan Tannous[a], Chi Zhang[b], and Doron Shabat[a]*

[a] School of Chemistry, Raymond and Beverly Sackler Faculty of Exact Sciences, Tel-Aviv University, Tel Aviv 69978 Israel.

[b] School of Chemistry and Chemical Engineering, Huazhong University of Science and Technology, Luoyu Road 1037, Wuhan 430074, China.

***Corresponding Author:**

Doron Shabat, Email: chdoron@tauex.tau.ac.il

Table of Contents

Synthetic procedures and characterization of caspase-3 chemiluminescent probe (Ac-DEVD-CL) 4

Protocols for measurements of caspase-3 activity in cancer cells .... Error! Bookmark not defined.

Cell culture protocol 7

Chemiluminescence cell imaging 7

Statistical Analysis 7

Supplementary figures 8

NMR and Mass spectra 12

HPLC spectra of key compounds 18

References 21

General methods

All reactions requiring anhydrous conditions were performed under an Argon atmosphere. All reactions were carried out at room temperature unless stated otherwise. Chemicals and solvents were either A.R. grade or purified by standard techniques. Thin layer chromatography (TLC): silica gel plates Merck 60 F254: compounds were visualized by irradiation with UV light. Column chromatography (FC): silica gel Merck 60 (particle size 0.040-0.063 mm), eluent given in parentheses. Reverse-phase high-pressure liquid chromatography (RP-HPLC): C18 5u, 250x4.6mm, eluent given in parentheses. Preparative RP-HPLC: C18 5u, 250x21mm, eluent given in parentheses. ^{1}H-NMR spectra were measured using Bruker Avance operated at 400MHz. ^{13}C-NMR spectra were measured using Bruker Avance operated at 100 MHz. Chemical shifts were reported in ppm on the δ scale relative to a residual solvent ($CDCl_3$: δ = 7.26 for ^{1}H-NMR and 77.16 for ^{13}C-NMR). Mass spectra were measured on Waters Xevo TQD. Chemiluminescence was recorded on Molecular Devices Spectramax iD3. Fluorescence was recorded on Tecan infinite 200 Pro. All chemicals, unless otherwise stated, were obtained from commercial sources. Light irradiation for photochemical reactions: LED PAR38 lamp (19W, 3000K). All general reagents, including salts and solvents, were purchased from Sigma-Aldrich. Caspase-3 (CASP3) Human produced in Sf9 Baculovirus cells was purchased from Prospec. Light irradiation for photochemical reactions: LED PAR38 lamp (19W, 3000K). Tripeptide was prepared by SPPS manually using a sintered glass filter tube. Fluorescence images of cells were acquired on Laser Scanning Microscope LSM800 (Zeiss).

Abbreviations

ACN- Acetonitrile, **DCM -** Dichloromethane, **DIPEA -** N,N-Diisopropylethylamine, **DMF -** N,N'-Dimethylformamide**,** **DMBA -** 1,3-Dimethylbarbituric acid, **DTT -** 1,4-dithiothreitol, **EtOAc -** Ethylacetate**, EEDQ -** N-Ethoxycarbonyl-2-ethoxy-1,2- dihydroquinoline, **Hex -** Hexanes, **HFIP -** Hexafluoro-2-propanol, **MB**- Methylene blue, **TES –** triethyl silane, **TFA -** Trifluoroacetic acid, **THF –** Tetrahydrofuran**, TMSCl -** Trimethylsilyl chloride, **HBTU -** Hexafluorophosphate benzotriazole tetramethyl uronium, **MB**- Methylene blue**.**

Synthetic procedures and characterization of the chemiluminescent Caspase-3 probe

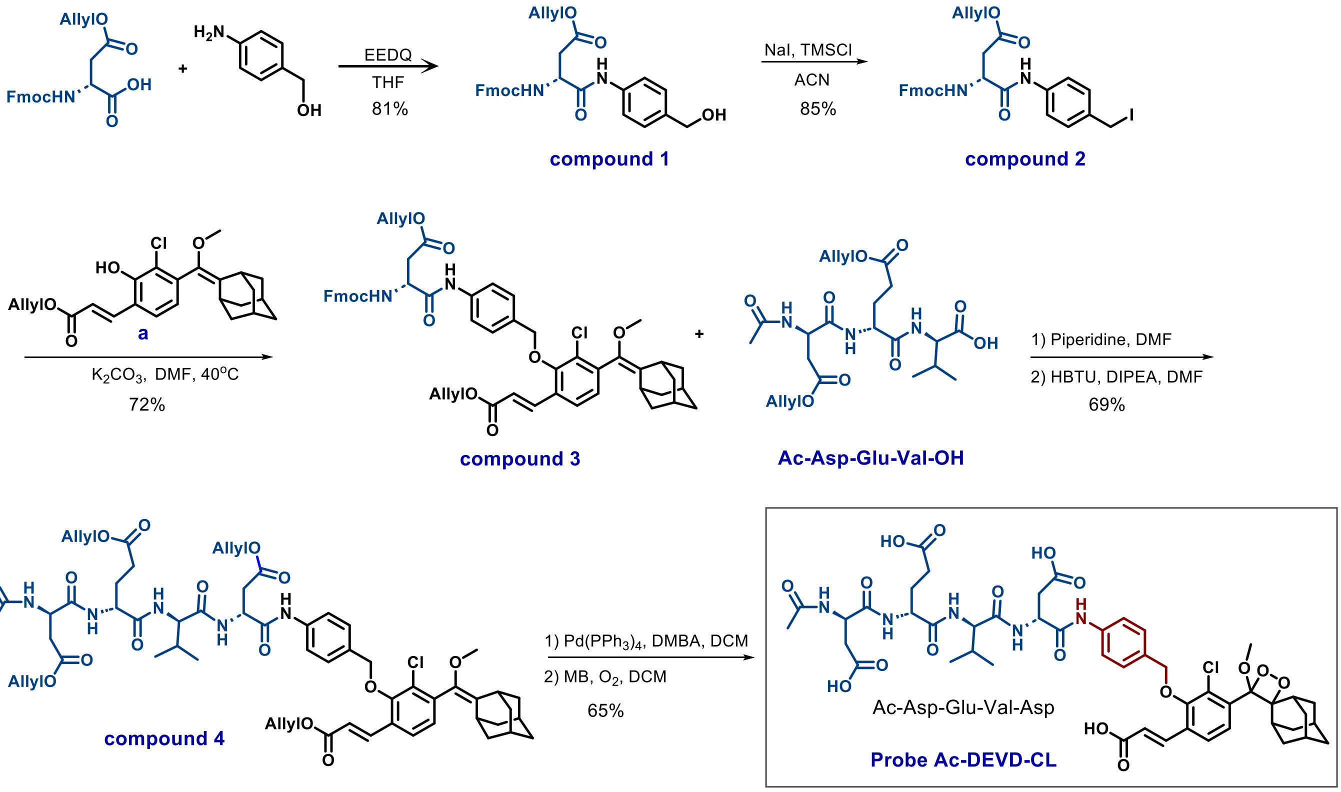

Scheme S1. Probe Ac-DEVD-CL synthesis

Compound 1

4-amino benzyl alcohol (100 mg, 0.81 mmol, 1 eq.) and Fmoc-L-aspartic acid 4-allyl ester (Fmoc-Asp(OAll)-OH) (353 mg, 0.89 mmol, 1.1 eq.) were dissolved in 2 ml of THF followed by the addition of EEDQ (301 mg, 1.22 mmol, 1.5 eq.). The reaction mixture was stirred at room temperature and monitored by TLC (70:30, EtOAc: Hex). Upon completion, the reaction mixture was diluted with EtOAc and washed with 1M HCl followed by brine. The organic layer was separated, dried over Na_2SO_4, filtered and the solvent was evaporated under reduced pressure. The crude product was further purified by column chromatography (70:30, EtOAc: Hex). to afford compound **1** in the form of a white solid (329 mg, 0.66 mmol, 81%).

^{1}H NMR (400 MHz, $CDCl_3$) δ 8.46 (s, 1H), 7.76 (d, *J* = 7.5 Hz, 2H), 7.58 (d, *J* = 7.4 Hz, 2H), 7.46 (d, *J* = 8.4 Hz, 2H), 7.40 (t, *J* = 7.4 Hz, 2H), 7.29 (dd, *J* = 7.4, 5.0 Hz, 4H), 6.11 (d, *J* = 7.8 Hz, 1H), 5.90 (ddd, *J* = 16.3, 11.0, 5.8 Hz, 1H), 5.33 (dd, *J* = 17.2, 1.3 Hz, 1H), 5.25 (dd, *J* = 10.4, 1.2 Hz, 1H), 4.70 (s, 1H), 4.67 – 4.58 (m, *J* = 7.0 Hz, 4H), 4.48 (d, *J* = 6.6 Hz, 2H), 4.22 (t, *J* = 6.7 Hz, 1H), 3.08 (d, *J* = 16.3 Hz, 1H), 2.78 (dd, *J* = 17.2, 6.3 Hz, 1H).

^{13}C NMR (100 MHz, $CDCl_3$) δ 171.99, 168.45, 156.53, 143.71, 143.65, 141.49, 137.36, 136.86, 131.58, 127.99, 127.89, 127.28, 125.08, 120.30, 120.23, 119.14, 67.50, 66.14, 65.04, 51.75, 47.26, 36.06.

MS (ES+): m/z calc. for $C_{29}H_{28}N_2O_6$:500.2; found: 523.6 $[M+Na]^+$.

Compound 2

Compound **1** (300 mg, 0.60 mmol, 1 eq.) was dissolved in 4 ml of ACN. Sodium Iodide (269 mg, 1.80 mmol, 3 eq.) was added followed by the rapid addition of TMS-Cl (227 µl, 1.80 mmol, 3 eq.). The reaction was monitored by TLC (50:50, EtOAc:Hex). Upon completion, the reaction mixture was diluted with EtOAc, and washed with saturated $Na_2S_2O_3$ followed by brine. The organic layer was separated, dried over Na_2SO_4, filtered and the solvent was evaporated under reduced pressure. The crude product was further purified by column chromatography (50:50, EtOAc: Hex) to afford compound **2** in the form of a yellowish solid (311 mg, 0.51 mmol, 85%).
^{1}H NMR (400 MHz, $CDCl_3$) δ 8.69 (s, 1H), 7.75 (d, *J* = 7.5 Hz, 2H), 7.59 – 7.52 (m, 2H), 7.43 (d, *J* = 8.5 Hz, 2H), 7.39 (t, *J* = 7.5 Hz, 2H), 7.32 – 7.24 (m, 4H), 6.23 (d, *J* = 8.2 Hz, 1H), 5.88 (dq, *J* = 11.0, 5.8 Hz, 1H), 5.31 (d, *J* = 17.1 Hz, 1H), 5.23 (dd, *J* = 10.4, 1.0 Hz, 1H), 4.78 (s, 1H), 4.61 (d, *J* = 5.4 Hz, 2H), 4.49 – 4.37 (m, 4H), 4.19 (t, *J* = 6.8 Hz, 1H), 3.03 (d, *J* = 14.1 Hz, 1H), 2.84 (dd, *J* = 16.8, 5.9 Hz, 1H).
^{13}C NMR (100 MHz, $CDCl_3$) δ 171.65, 168.73, 156.62, 143.70, 141.47, 137.12, 135.61, 131.65, 129.60, 128.00, 127.30, 125.13, 120.44, 120.24, 119.08, 67.62, 66.11, 51.96, 47.21, 36.31, 5.83.
MS (ES+): m/z calc. for $C_{29}H_{27}IN_2O_5$:610.1; found: 633.6 $[M+Na]^+$.

Compound 3

Phenol enol ether **a**[1] (100 mg, 0.24 mmol, 1.1 eq.) and K_2CO_3 (45 mg, 0.33 mmol, 1.5 eq.) were dissolved in 2 mL of dry DMF. The solution was stirred for 5 minutes, then compound **2** (134 mg, 0.22 mmol, 1 eq.) was added. The reaction mixture was stirred at 40°C and monitored by TLC (50:50, EtOAc:Hex). Upon completion, the reaction mixture was diluted with EtOAc and washed with 1M HCl followed by brine. The organic layer was separated, dried over Na_2SO_4, and evaporated under reduced pressure. The crude product was purified by column chromatography on silica gel (50:50, EtOAc:Hex) to afford compound **3** in the form of a white solid (144 mg, 0.16 mmol, 72%).
^{1}H NMR (400 MHz, $CDCl_3$) δ 8.50 (s, 1H), 7.95 (d, *J* = 16.2 Hz, 1H), 7.77 (d, *J* = 7.5 Hz, 2H), 7.59 (d, *J* = 7.4 Hz, 2H), 7.51 (d, *J* = 8.5 Hz, 2H), 7.46 – 7.37 (m, 5H), 7.30 (t, *J* = 7.4 Hz, 2H), 7.07 (d, *J* = 8.0 Hz, 1H), 6.46 (d, *J* = 16.2 Hz, 1H), 6.10 (d, *J* = 7.0 Hz, 1H), 6.04 – 5.95 (m, 1H), 5.94 – 5.85 (m, 1H), 5.40 – 5.31 (m, 2H), 5.30 – 5.23 (m, 2H), 4.97 (d, *J* = 3.6 Hz, 2H), 4.74 – 4.69 (m, 3H), 4.64 (d, *J* = 5.7 Hz, 2H), 4.50 (d, *J* = 6.2 Hz, 2H), 4.23 (t, *J* = 6.7 Hz, 1H), 3.32 (s, 3H), 3.28 (s, 1H), 3.10 (d, *J* = 16.2 Hz, 1H), 2.79 (dd, *J* = 17.0, 5.9 Hz, 1H), 2.07 (s, 1H), 2.00 – 1.62 (m, 12H).
^{13}C NMR (100 MHz, $CDCl_3$) δ 172.01, 168.38, 166.39, 156.54, 153.79, 143.71, 143.68, 141.50, 139.56, 139.11, 138.34, 137.77, 132.55, 132.38, 132.32, 131.59, 129.99, 129.88, 129.82, 127.99, 127.93, 127.28, 125.21, 125.09, 120.23, 120.12, 120.04, 119.14, 118.41, 75.79, 67.52, 66.15, 65.42, 57.39, 51.79, 47.29, 39.34, 39.19, 38.76, 37.20, 36.08, 33.08, 31.73, 29.84, 28.50, 28.35, 22.79, 14.26.
MS (ES+): m/z calc. for $C_{53}H_{53}ClN_2O_9$: 896.3; found:919.9 $[M+Na]^+$.

Ac-Asp-Glu-Val-OH

The tripeptide Ac-Asp-Glu-Val-OH was synthesized using standard solid-phase peptide synthesis (SPPS) protocols on a 2-chlorotrityl chloride resin. The resin (500 mg, 1.60 mmol/g, 0.8 mmol) was swollen in dichloromethane (DCM, 5 mL) for 20 minutes. Fmoc-Val-OH (2 equiv.) was coupled to the resin using DIPEA (4 equiv.) in DCM, and the reaction mixture was agitated for 2 hours. After coupling, unreacted sites on the resin were capped using a solution of DCM, methanol, and DIPEA (17:2:1) for 30 minutes. The resin was washed sequentially with DMF and DCM (twice each). The Fmoc protecting group was removed by treating the resin with 20% piperidine in DMF for two cycles of 5 and 10 minutes, followed by thorough washing with DMF (three times) and DCM (three times). Subsequently, Fmoc-Glu(OAll)-OH (4 equiv.) was activated with HBTU (4 equiv.) and DIPEA (6 equiv.) in DMF for 5 minutes and coupled to the resin. The reaction proceeded for 30 minutes, after which the resin was washed with DMF and DCM

(three times each). The Fmoc group on the glutamic acid residue was removed under the same deprotection conditions as described above, followed by sequential washing with DMF (three times) and DCM (three times). Fmoc-Asp(OAll)-OH (4 equiv.) was then activated with HBTU (4 equiv.) and DIPEA (6 equiv.) in DMF for 5 minutes and added to the resin. After coupling for 30 minutes, the resin was washed with DMF (twice) and DCM (twice).
The N-terminal Fmoc group was removed using 20% piperidine in DMF for two cycles of 5 and 10 minutes. The resulting free amine was acetylated with a solution of acetic anhydride (10 equiv.) and DIPEA (10 equiv.) in DMF. The final peptide was cleaved from the resin using a cleavage cocktail of HFIP, TES, and DCM (50:5:45), followed by precipitation in Et_2O at 0°C.
MS (ES+): m/z calc. for $C_{22}H_{33}N_3O_9$: 483.2; found:506.7 $[M+Na]^+$.

Compound 4

Compound **3** (60 mg, 0.07 mmol, 1 eq) and piperidine (200 µL) were dissolved in DMF (1 mL). The solution was stirred for 30 minutes at room temperature and monitored by RP-HPLC (ACN in H_2O containing 0.1% TFA; gradient from 30 to 100%). After full deprotection of the Fmoc was observed the reaction was diluted with EtOAc was washed with saturated ammonium chloride followed with brine. The organic layer was separated, dried over Na_2SO_4 and evaporated under reduced pressure. Then, the crude was added to a premixed DMF (1 mL) solution containing tripeptide (32 mg, 0.07 mmol, 1 eq), HBTU (37.5 mg, 0.1 mmol, 1.5 eq), and DIPEA (34 µL, 0.20 mmol, 3 eq). The reaction was stirred at room temperature and monitored by (ACN in H_2O containing 0.1% TFA; gradient from 50 to 100%). Upon completion, the solvent was removed under reduced pressure and the crude was purified by preparative RP-HPLC (50-100%, ACN in H_2O with 0.1% TFA) to obtain compound **4** as white solid (52 mg, 0.04 mmol, 69%).
MS (ES+): m/z calc. for $C_{60}H_{74}ClN_5O_{15}$: 1139.5; found:1163.3 $[M+Na]^+$.

Probe Ac-DEVD-CL

Compound **4** (52 mg, 0.04 mmol) was dissolved in DCM (1 mL) and was treated with DMBA (31 mg, 0.2 mmol, 5 eq) and $Pd(PPh_3)_4$ (13 mg, 0.01 mmol). The reaction was stirred at room temperature and monitored by RP-HPLC (50-100%, ACN in H_2O with 0.1% TFA). Upon complete deprotection of the allyl-protecting groups, MB (catalytic amount) was added to the reaction mixture. Oxygen was bubbled through the solution and subjected to irradiation with yellow light. The reaction was monitored by RP-HPLC (50-100%, ACN in H_2O with 0.1% TFA), and upon completion the solvent was removed under reduced pressure, and the product was purified by preparative RP-HPLC (ACN in H_2O containing 0.1% TFA; gradient from 50 to 100%; flow rate: 20 mL/min).
Probe Ac-DEVD-CL was obtained as a white solid (25 mg, 0.02 mmol, 65% yield).
MS (ES-): m/z calc. for $C_{48}H_{58}ClN_5O_{17}$: 1011.4; found:1011.0 $[M-H]^-$.

Protocols for measurements of Caspase-3 activity in cancer cells

Cell culture protocol

Mouse breast cell line 4T1 cells were purchased from the American Type Culture Collection (ATCC). 4T1 cells were cultured in DMEM (Dulbecco's modified Eagle medium) (GIBCO) with 10% FBS (fetal bovine serum) (GIBCO) in a S7 humidified environment at 37 °C which contains 5% CO_2 and 95% air.

Chemiluminescence cell imaging

4T1 cells with the population of 2.5×10^4 cells were seeded into u-Slide 8 Well confocal cell culture dishes and incubated for 24 h. After 24 h incubation, the cells were replaced with fresh medium containing cisplatin (Cisp, 300 µM) with or without Caspase-3 Inhibitor I (CASP3i, 100 µM) for 3 hours. Afterward, probe Ac-DEVD-CL (10 µM) was added into all the wells and incubated for 20 minutes (0.1% v/v of DMSO). The old medium was removed, and the cells were washed with fresh medium 3 times. The chemiluminescence images of all the cells were recorded with LX71 inverted microscope (Olympus) with an infinity 3-1 (Lumenera) CCD camera. The excitation light filter was completely blocked during the imaging process and the chemiluminescence signals were recorded under an open filter with an acquisition period of 5 seconds.

Statistical Analysis

The data were expressed as mean ± SD. Statistical significance was calculated via one-way ANOVA with a Tukey post-hoc test. The data were classified with p-values and denoted by (*) for $p < 0.05$, (**) for $p < 0.01$, and (***) for $p < 0.001$.

Supplementary figures

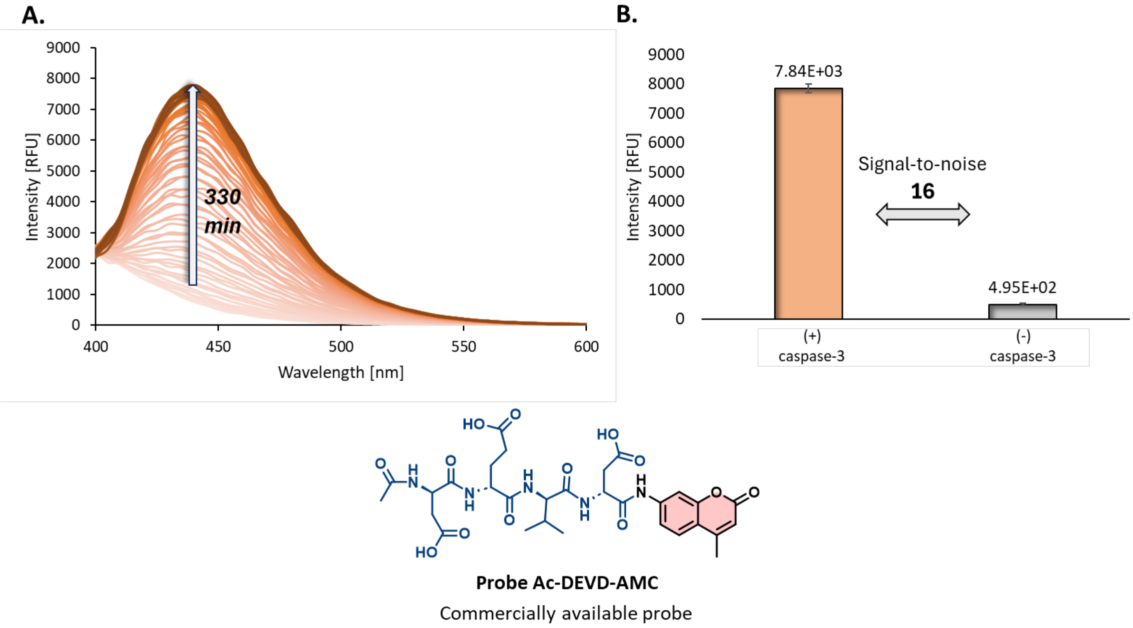

Figure S1. Fluorescence emission spectra during time (**A.**), and light intensity (**B.**) of the fluorescent caspase-3 commercial probe (Ac-DEVD-AMC) [50 µM] in the presence and absence of caspase-3 [1.66 ug/mL] in HEPES buffer (pH 7.5, 1% DMSO, 1mM DTT) at 37°C. $\lambda_{ex} = 350nm$.

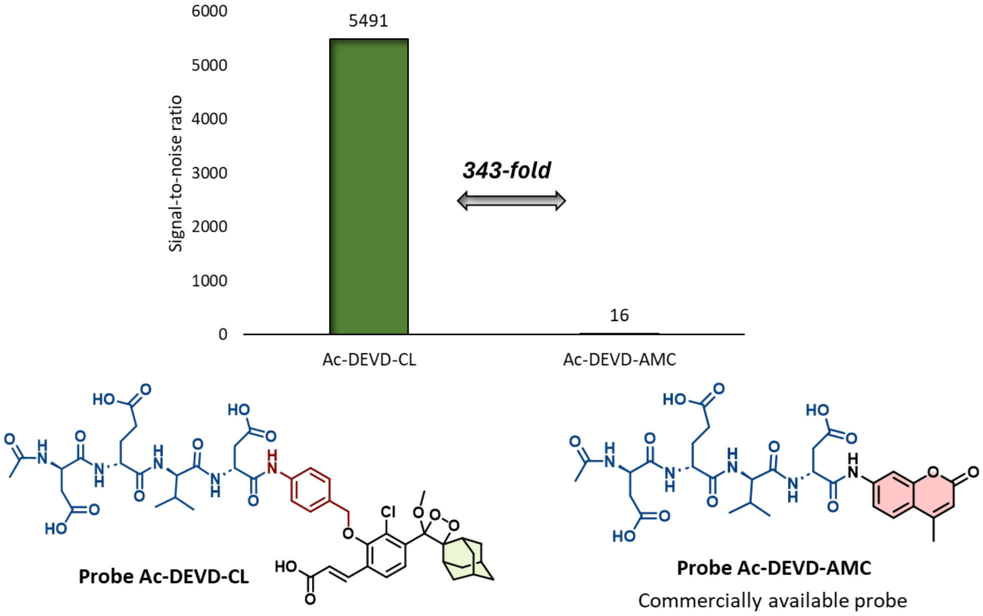

Figure S2. Signal-to-noise ratio comparison between caspase-3 chemiluminescent probe (Ac-DEVD-CL) [10µM] and caspase-3 commercially available fluorescent probe (Ac-DEVD-AMC) [50µM] in the presence and absence of caspase-3 [1.66 ug/mL] in HEPES buffer (pH 7.5, 1% DMSO, 1mM DTT) at 37°C. $\lambda_{ex} = 350nm$.

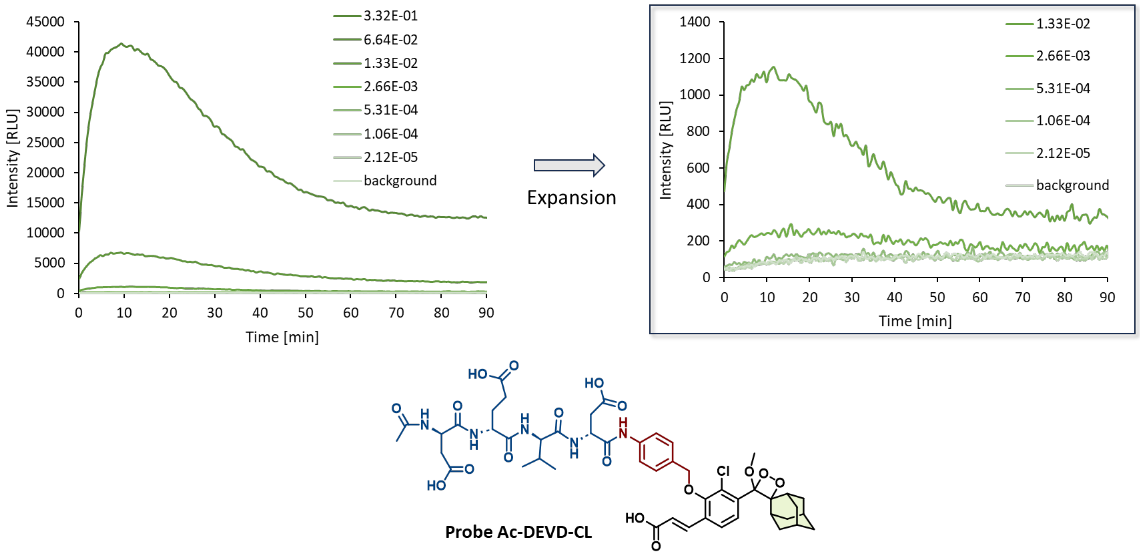

Figure S3. Chemiluminescent kinetic profiles for 90 minutes hours of probe Ac-DEVD-CL [10 µM] with various concentrations of caspase-3 [1.66-2.12×10^{-5} µg/mL] in HEPES buffer (pH 7.5, 1% DMSO, 1mM DTT) at 37°C.

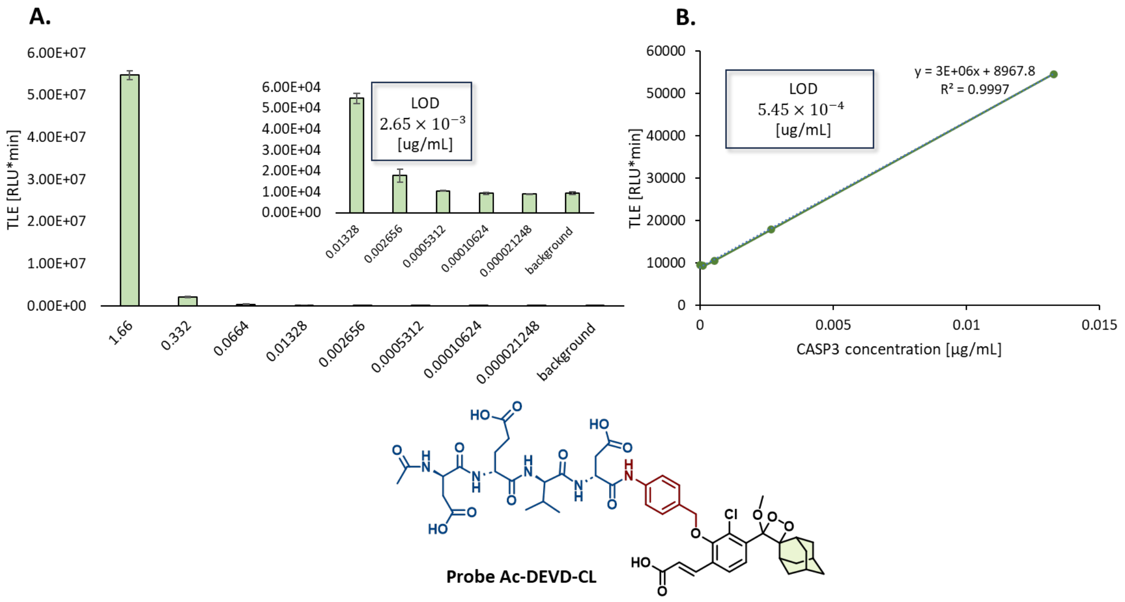

Figure S4. Total light emitted **(A.)** and linear calibration curve **(B.)** after 90 min of Ac-DEVD-CL probe [10 µM] with various concentrations of caspase-3 [1.66-2.12×10^{-5} µg/mL] in HEPES buffer (pH 7.5, 1% DMSO, 1mM DTT) at 37°C. The limit of detection (LOD) was determined using two methods: the blank + 3SD (standard deviation) method (left), and secondly, by a linear calibration curve. For the latter, the limit of detection is defined as 3 times the standard deviation of the blank divided by the slope of the linear calibration curve (LOD = 3σ/k) (right). Since the second method (LOD = 3σ/k) is more commonly used, the results of this LOD comparison are presented in the manuscript.

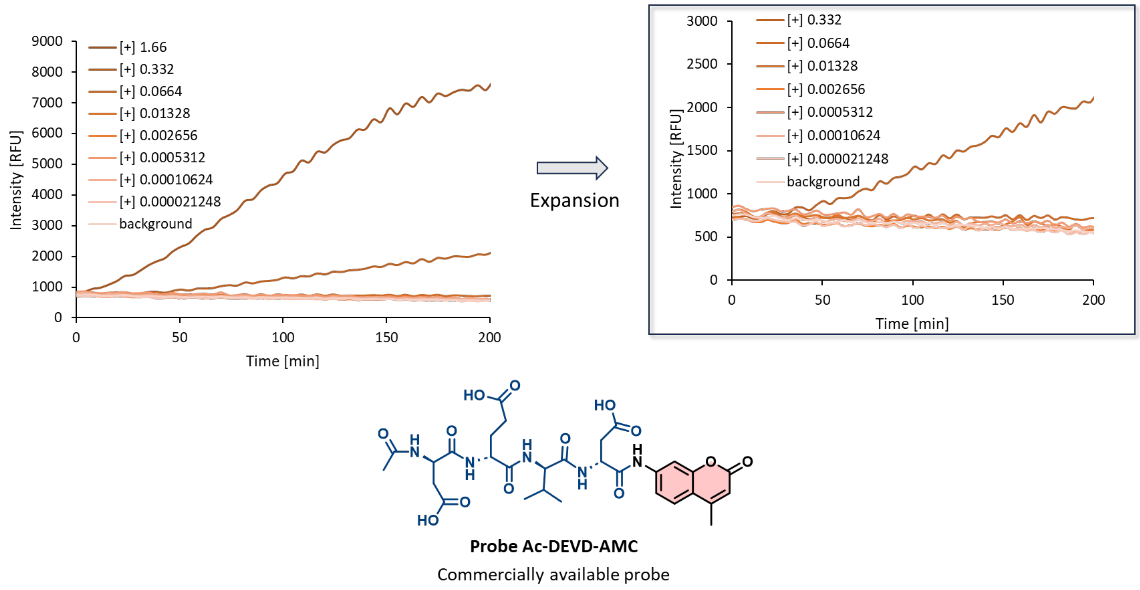

Figure S5. Fluorescent kinetic profiles during 200 minutes of Ac-DEVD-AMC probe [50 µM] with various concentrations of caspase-3 [1.66-2.12×10^{-5} µg/mL] in HEPES buffer (pH 7.5, 1% DMSO, 1mM DTT) at 37°C. ($\lambda_{ex} = 350nm, \lambda_{em} = 440nm$).

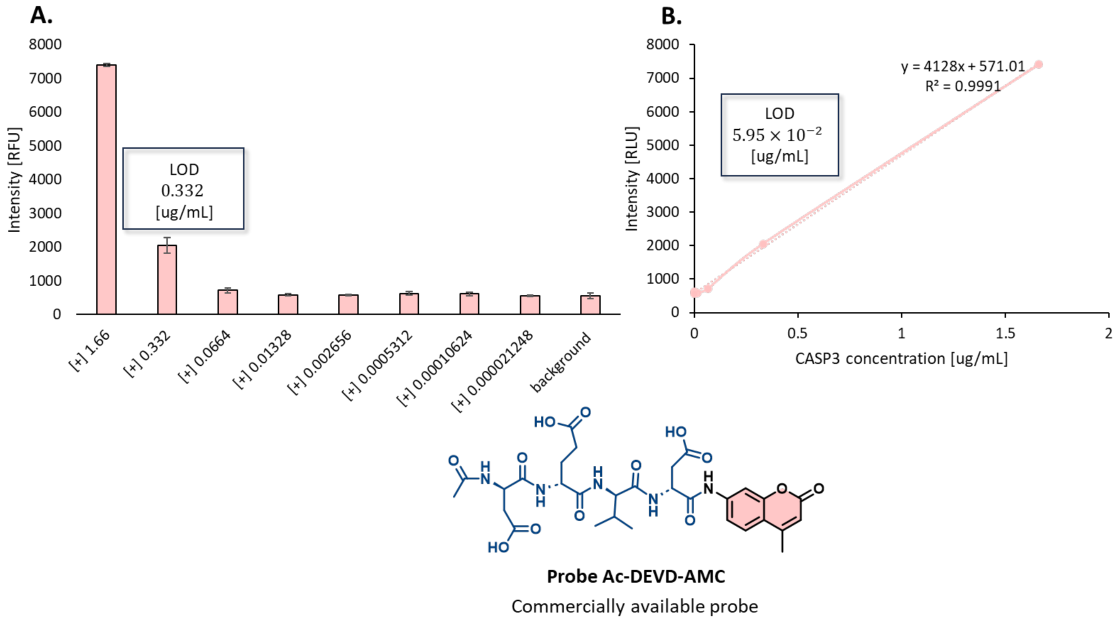

Figure S6. Total light emitted **(A.)** and linear calibration curve **(B.)** after 90 min of Ac-DEVD-AMC probe [50 µM] with various concentrations of caspase-3 [1.66-2.12×10^{-5} µg/mL] in HEPES buffer (pH 7.5, 1% DMSO, 1mM DTT) at 37°C.

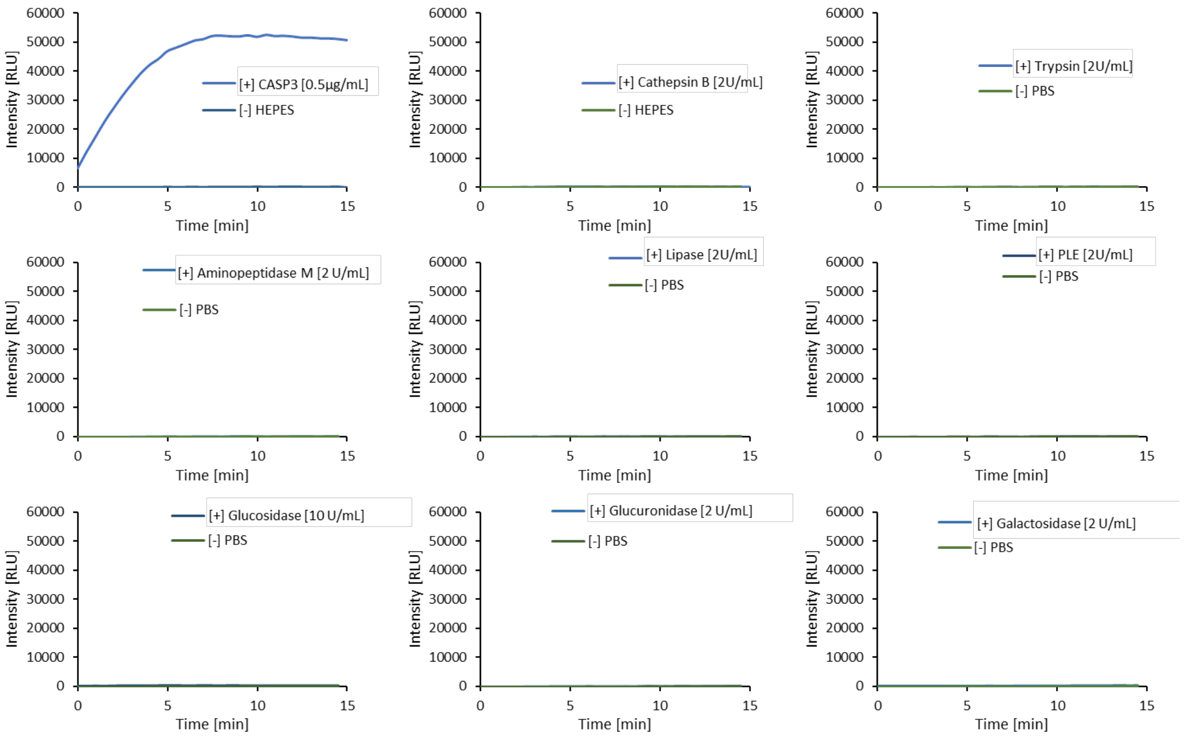

Figure S7. The selectivity of the chemiluminescent CASP3 probe (Ac-DEVD-CL) [10 µM in HEPES buffer (pH 7.5), 1% DMSO, 1mM DTT, 37°C] was evaluated in the presence of 9 commercially available recombinant enzymes. The chemiluminescent intensity was measured in the presence of each of the following commercially available recombinant enzymes: CASP3 Human (from Sf9 Baculovirus cells) [0.5 µg/mL], Cathepsin B (from human liver) [2U/mL], Trypsin (from porcine pancreas) [2U/mL], Aminopeptidase-M (from Porcine Kidney) [2 U/mL], Lipase (from Pseudomonas cepacia) [2 U/mL], Esterase (from porcine liver) [2U/mL], β-glucosidase (from almonds) [10 U/mL], β-glucuronidase (from *E. coli*) [2 U/mL], β-galactosidase (from *E. coli*) [2 U/mL].

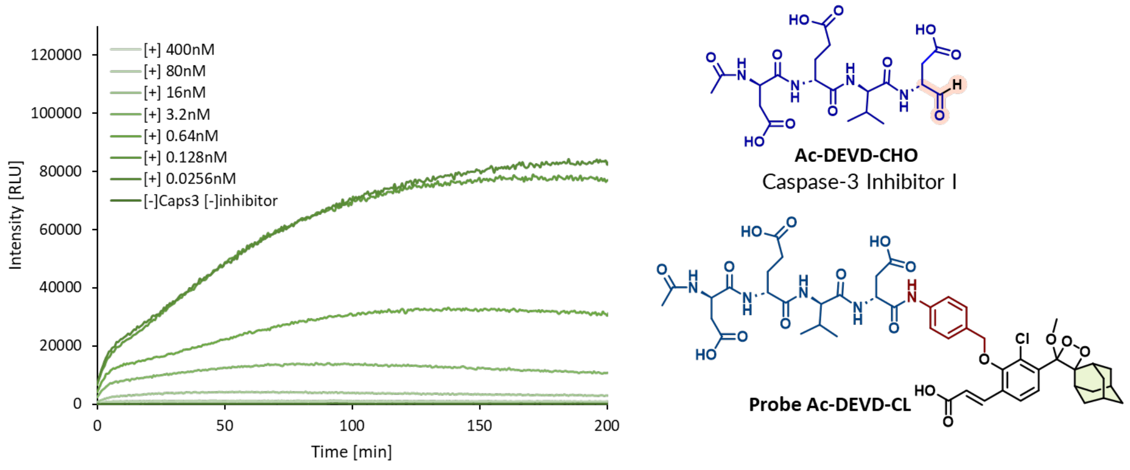

Figure S8. Chemiluminescent kinetic profile during 200 minutes of **Ac-DEVD-CL** [10µM] in the presence of various concentration of caspase-3 inhibitor I [400-0.0256 nM] with caspase-3 [0.15ug/mL] in HEPES buffer (pH 7.5, 1% DMSO, 1mM DTT) at 37°C.

NMR spectra

Compound 1

^{1}H-NMR

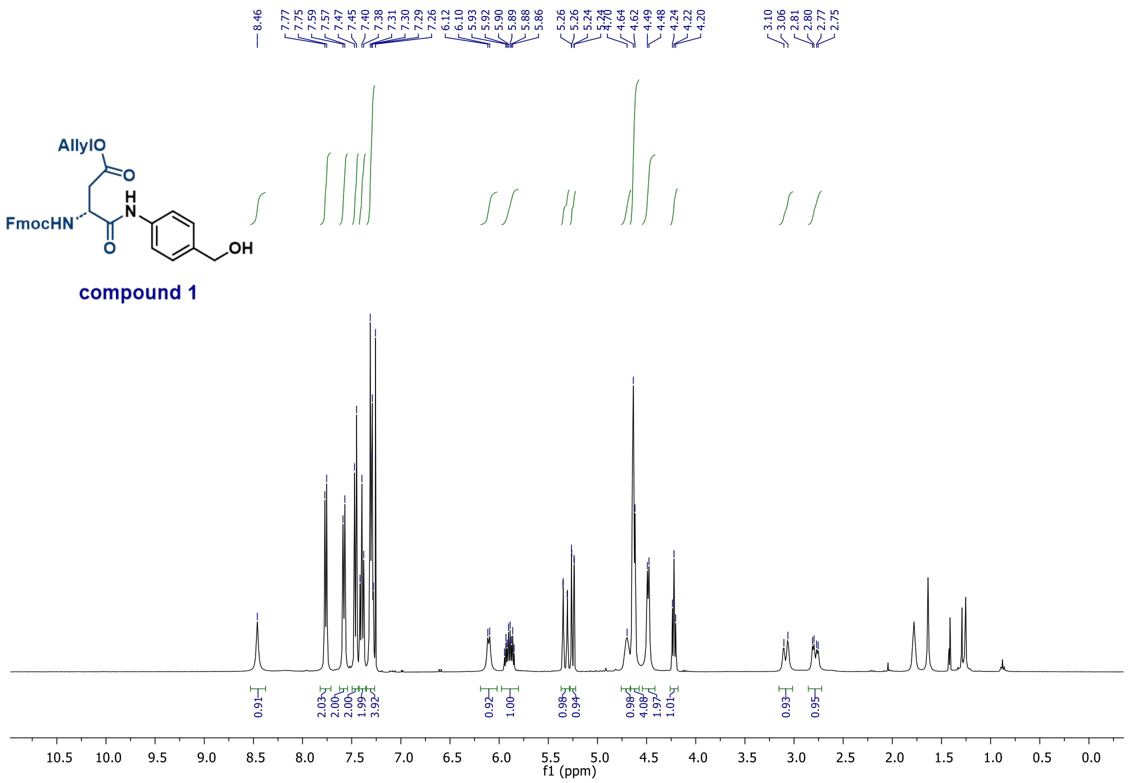

^{13}C-NMR

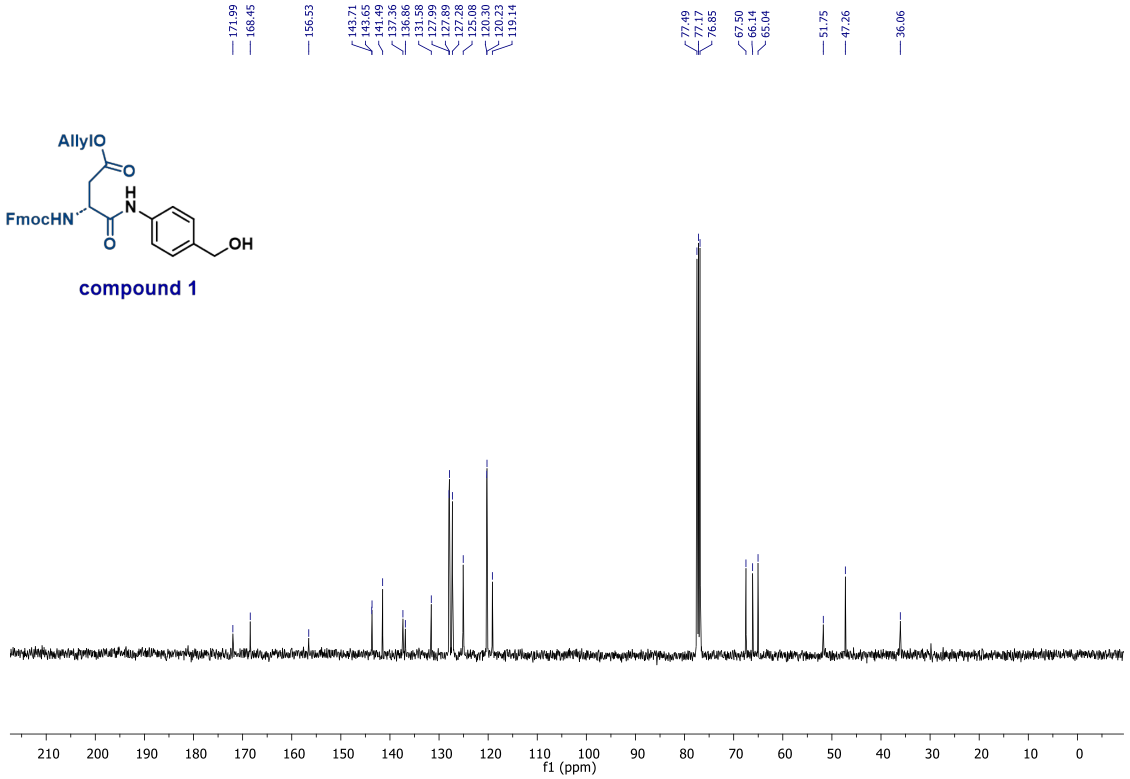

Mass spectra

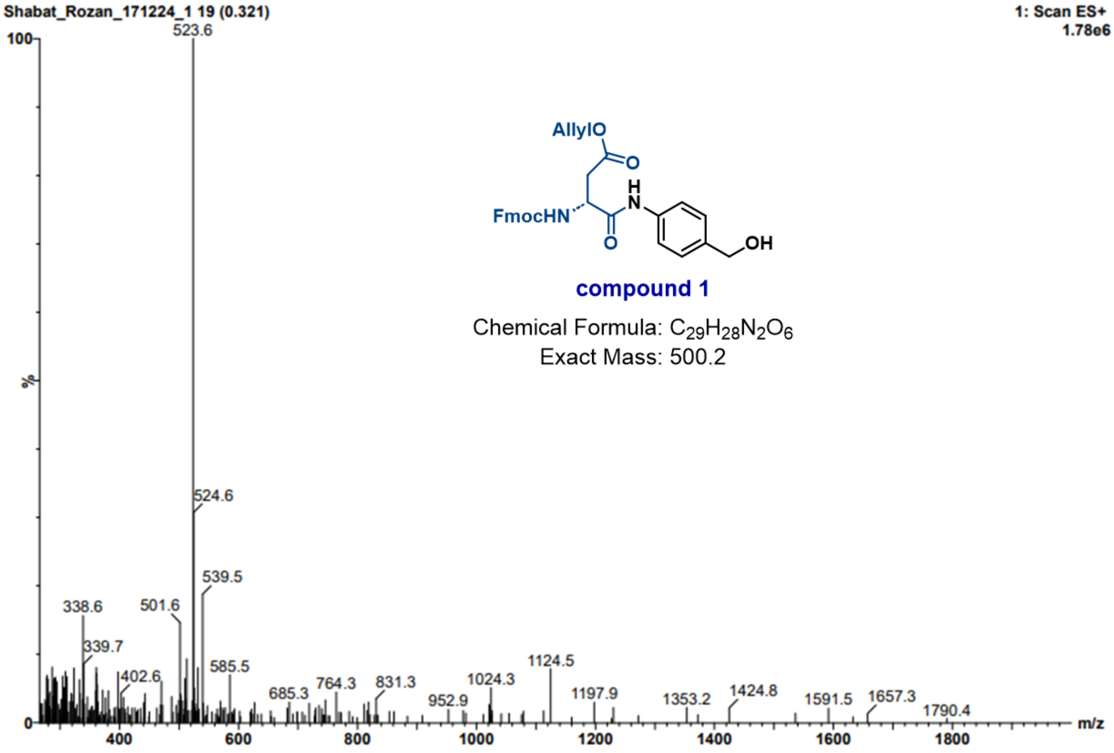

Compound 2

^{1}H-NMR

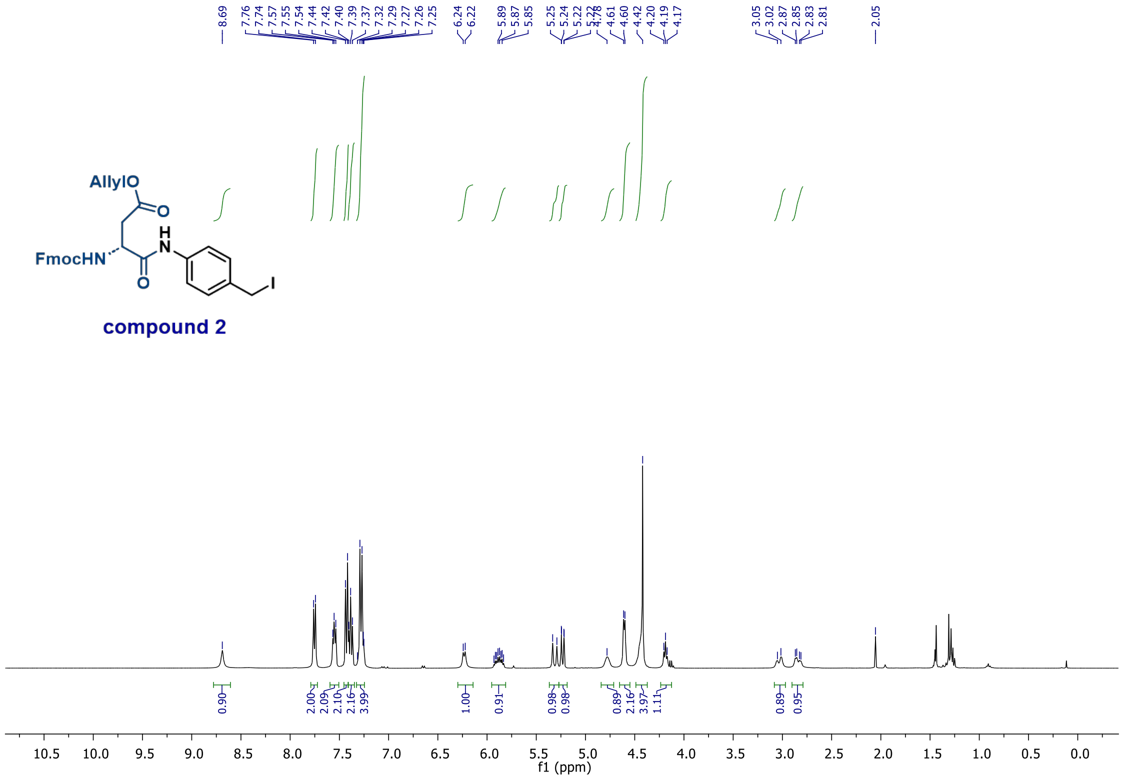

^{13}C-NMR

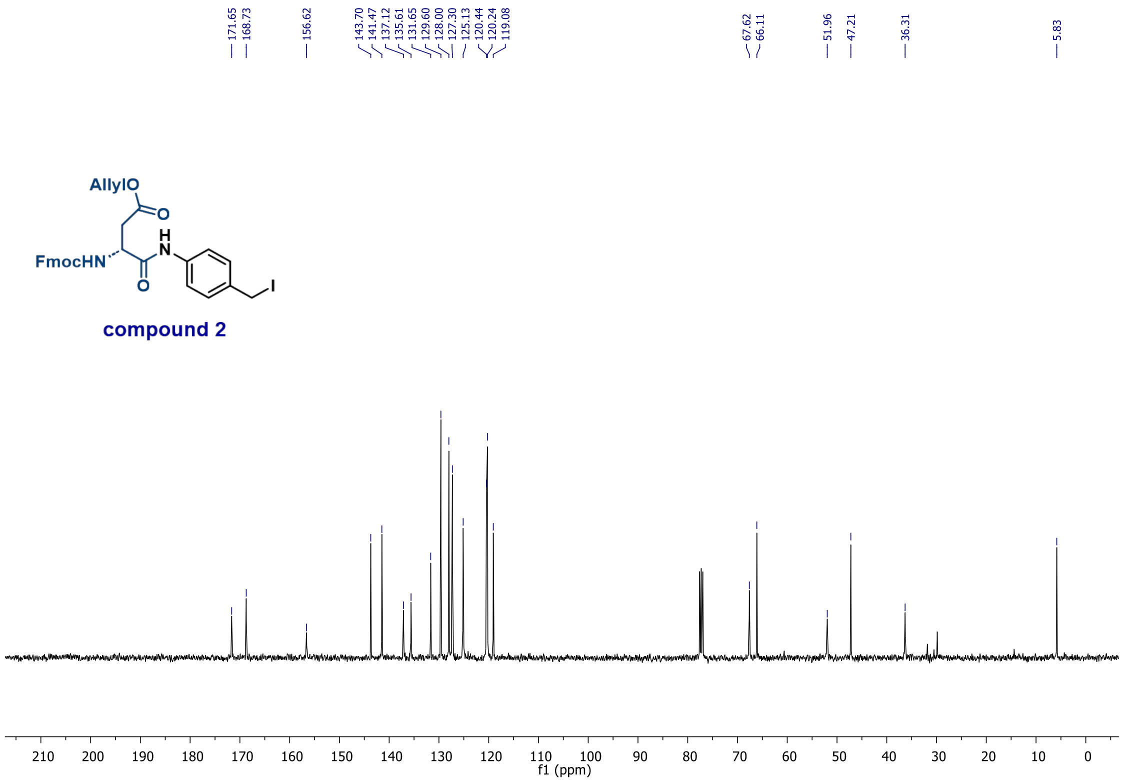

Mass spectra

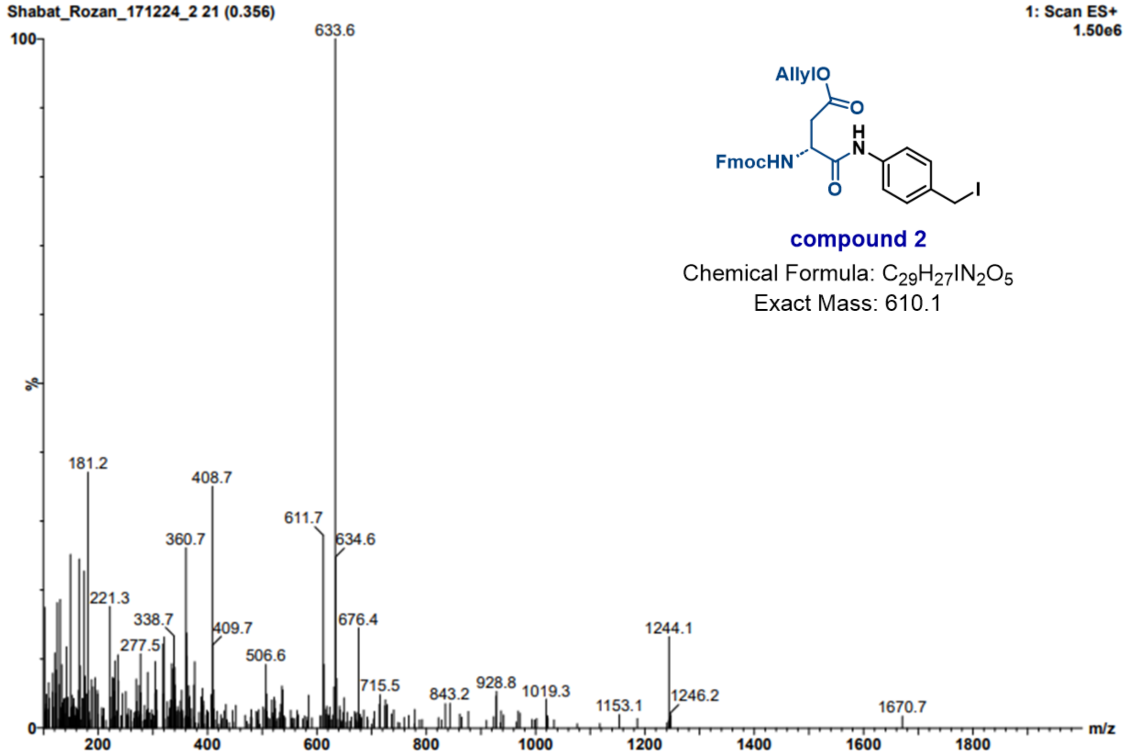

Compound 3

^{1}H-NMR

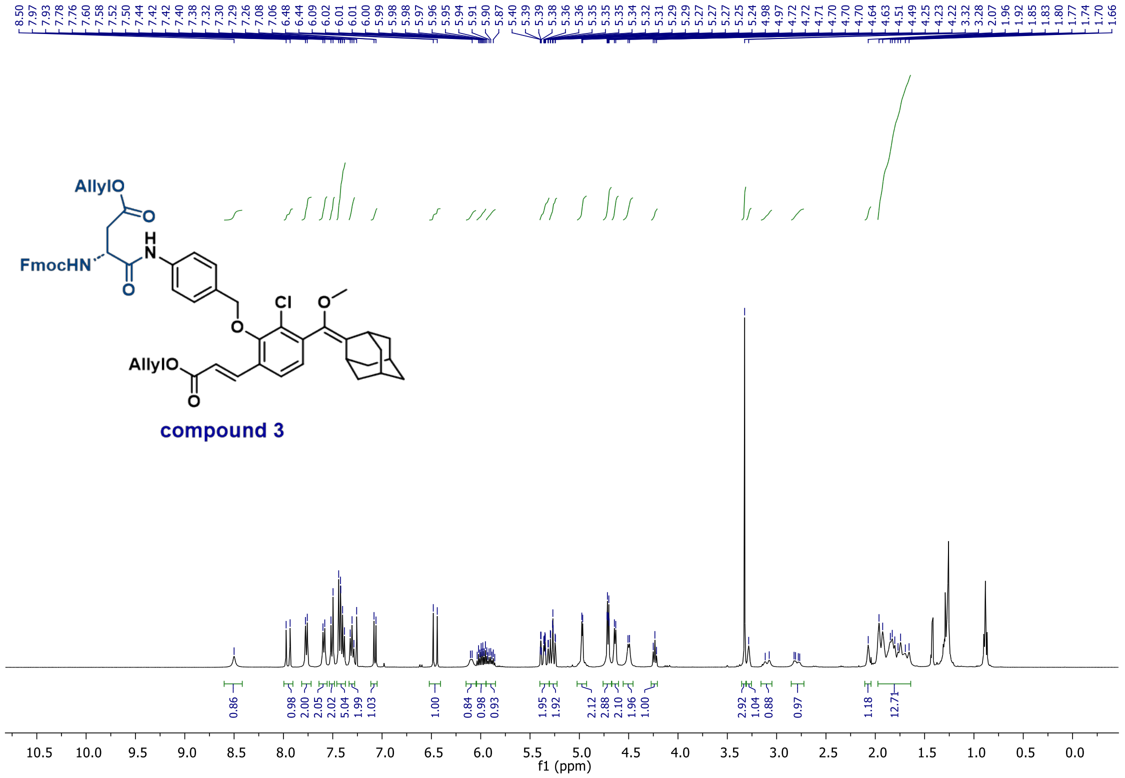

^{13}C-NMR

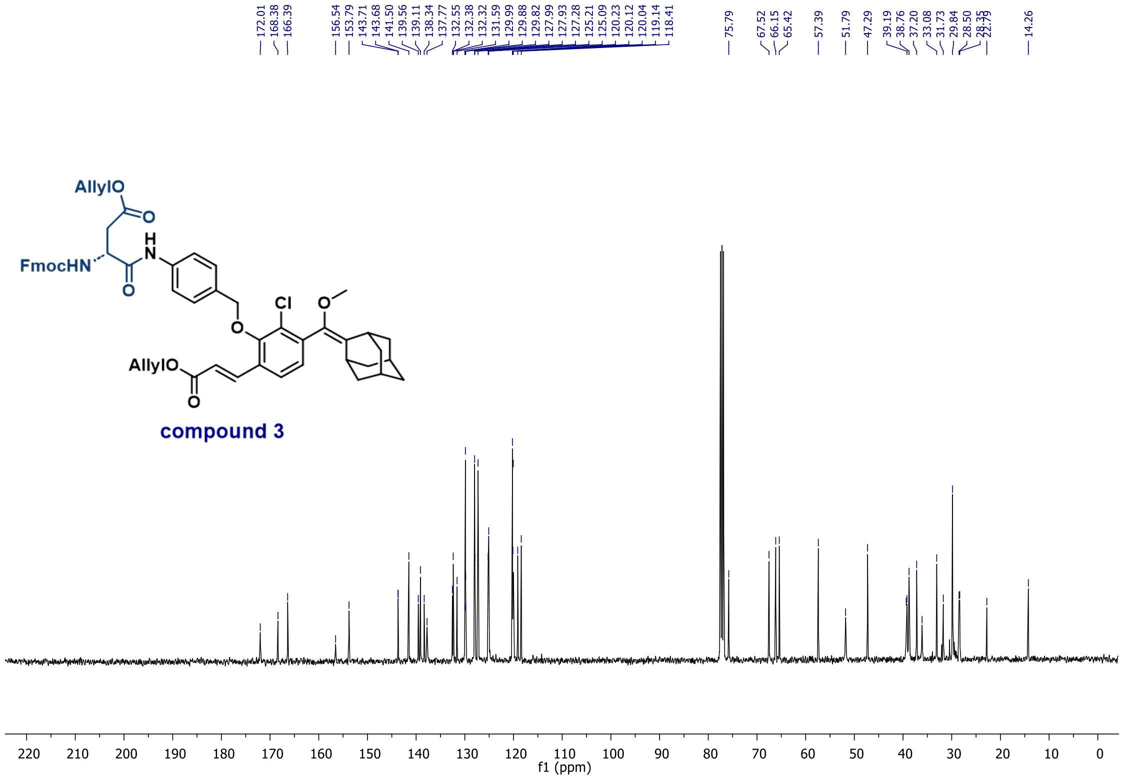

Mass spectra

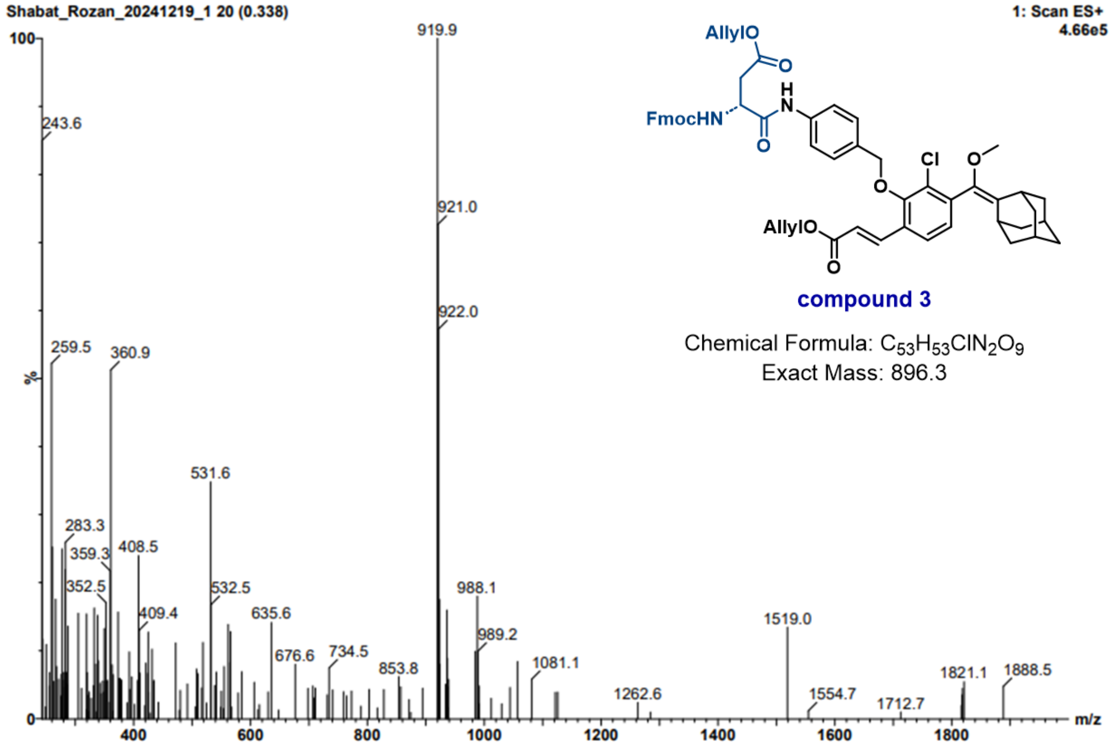

Ac-Asp-Glu-Val-OH

Mass spectra

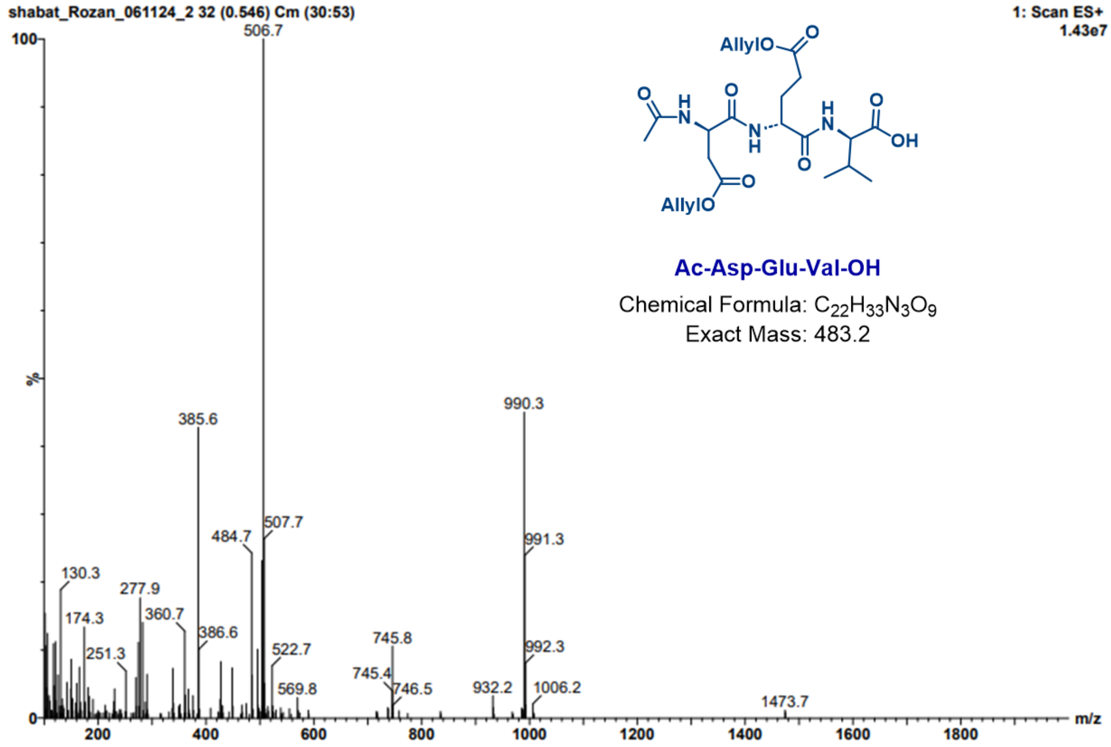

Compound 4

Mass spectra

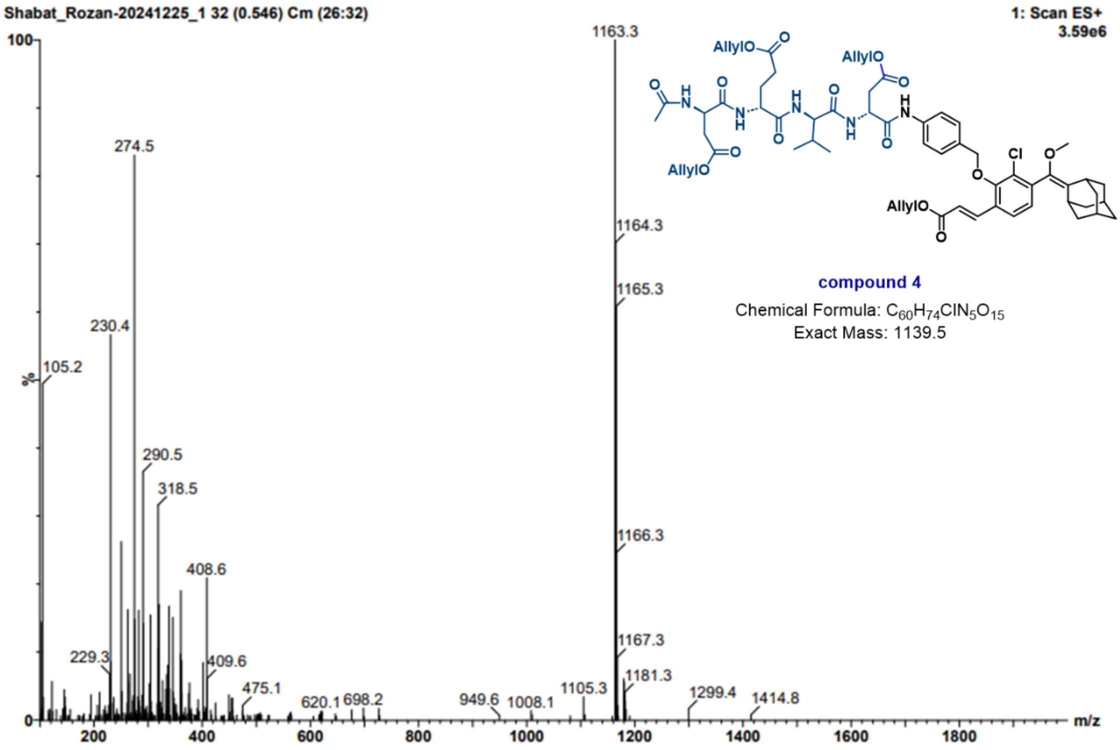

Probe Ac-DEVD-CL

Mass spectra

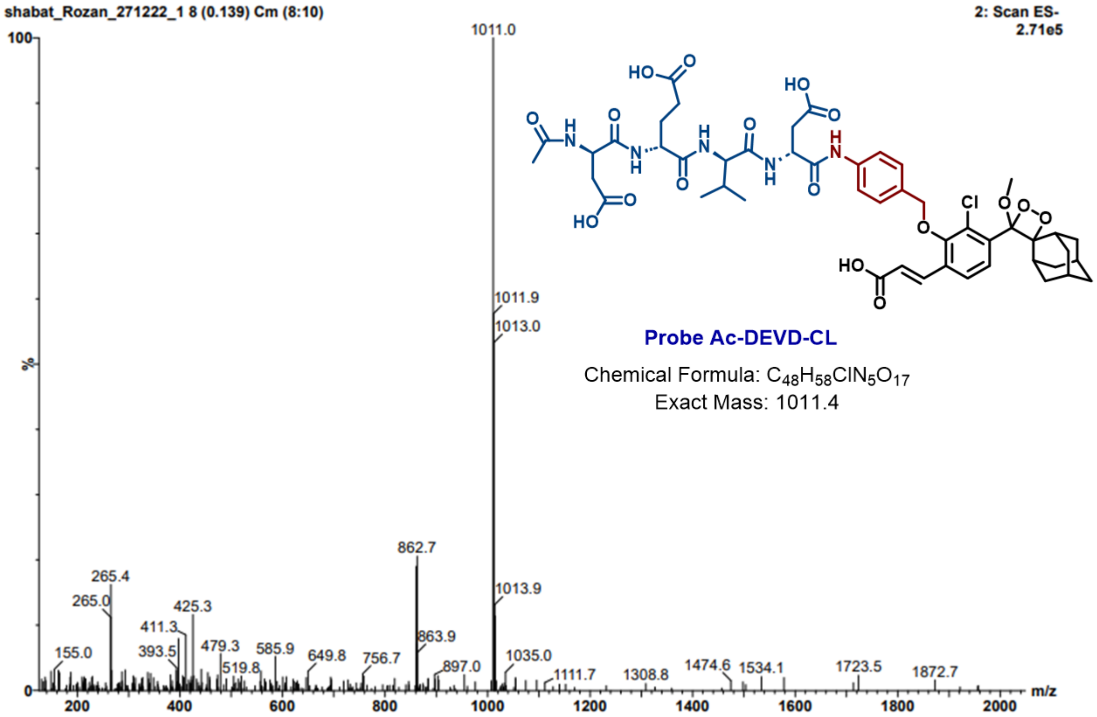

HPLC spectra of key compounds

Compound 3

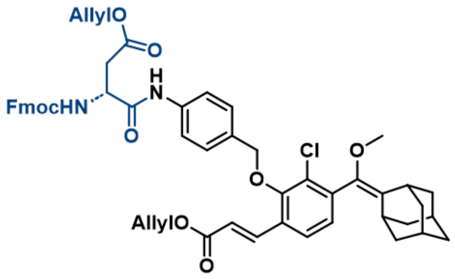

compound 3

3D HPLC spectra (50-100% ACN in water, 0.1%TFA)

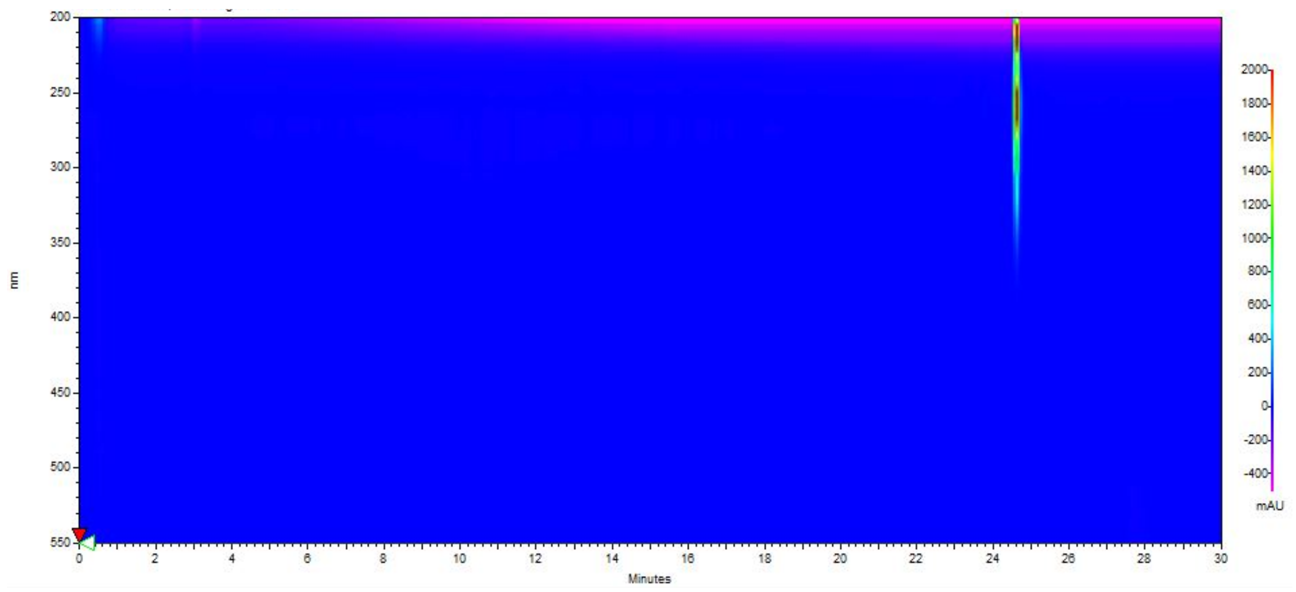

2D HPLC spectra (Absorbance measured at 270nm)

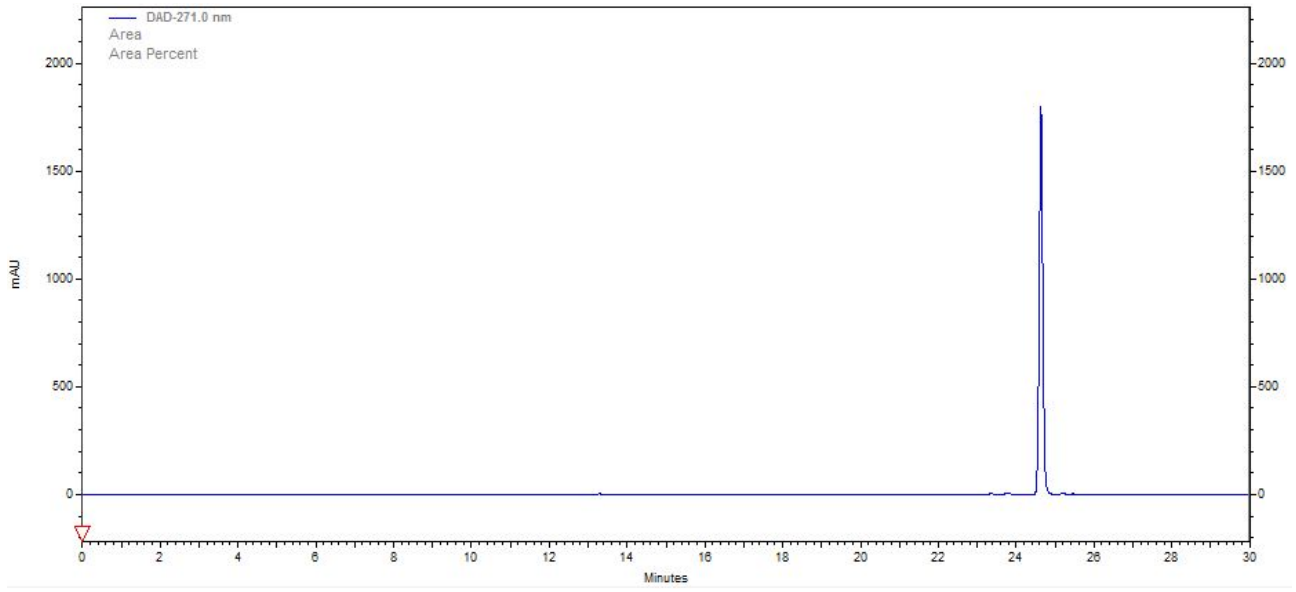

Compound 4

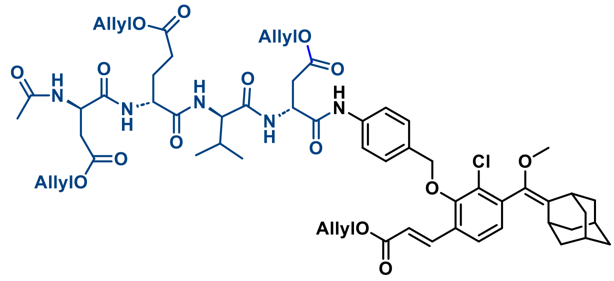

compound 4

3D HPLC spectra (70-100% ACN in water, 0.1%TFA)

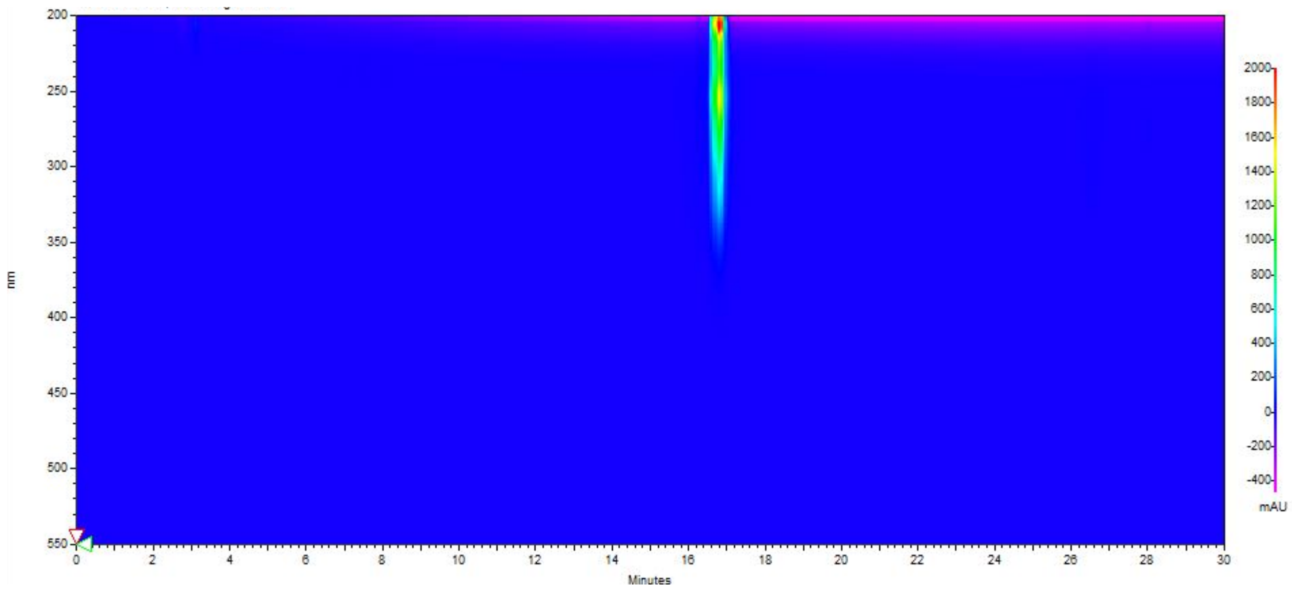

2D HPLC spectra (Absorbance measured at 270nm)

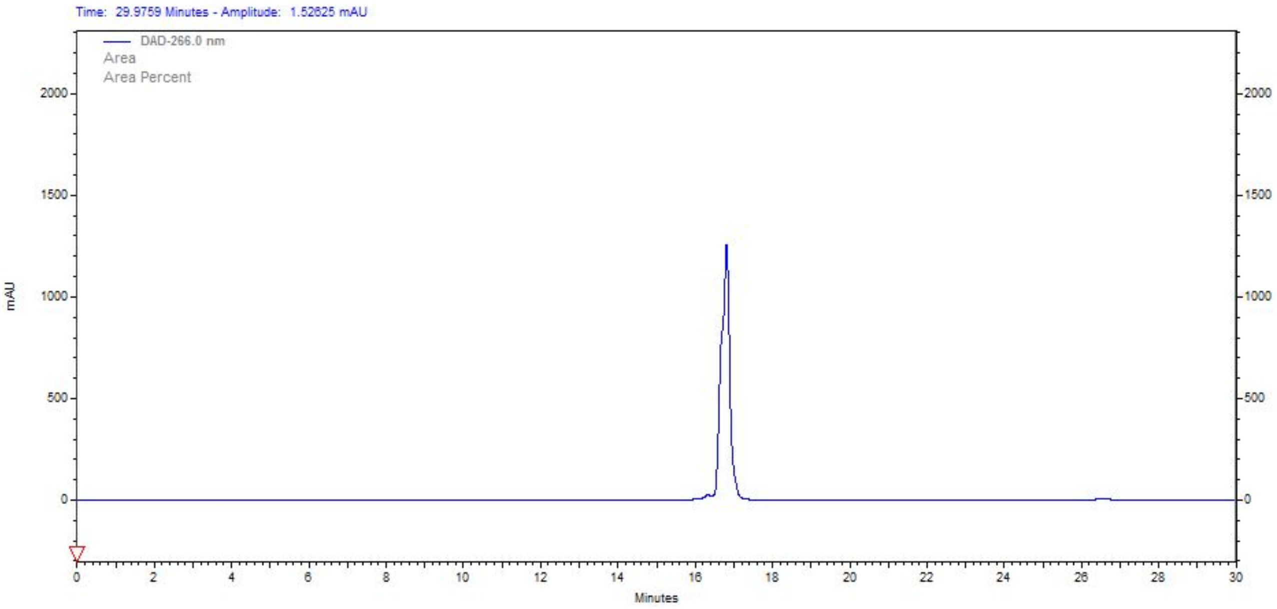

Probe Ac-DEVD-CL

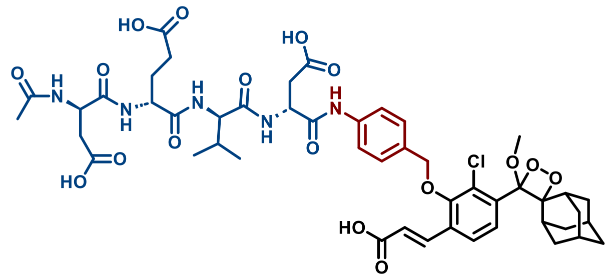

Probe Ac-DEVD-CL

3D HPLC spectra (50-100% ACN in water, 0.1%TFA)

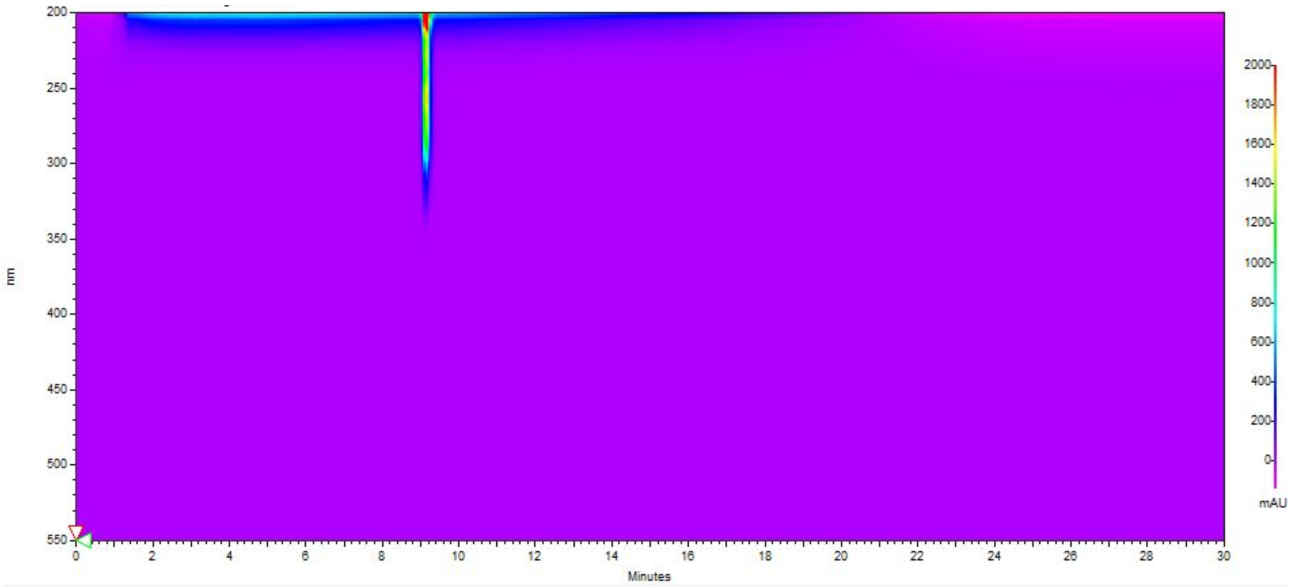

2D HPLC spectra (Absorbance measured at 270nm)

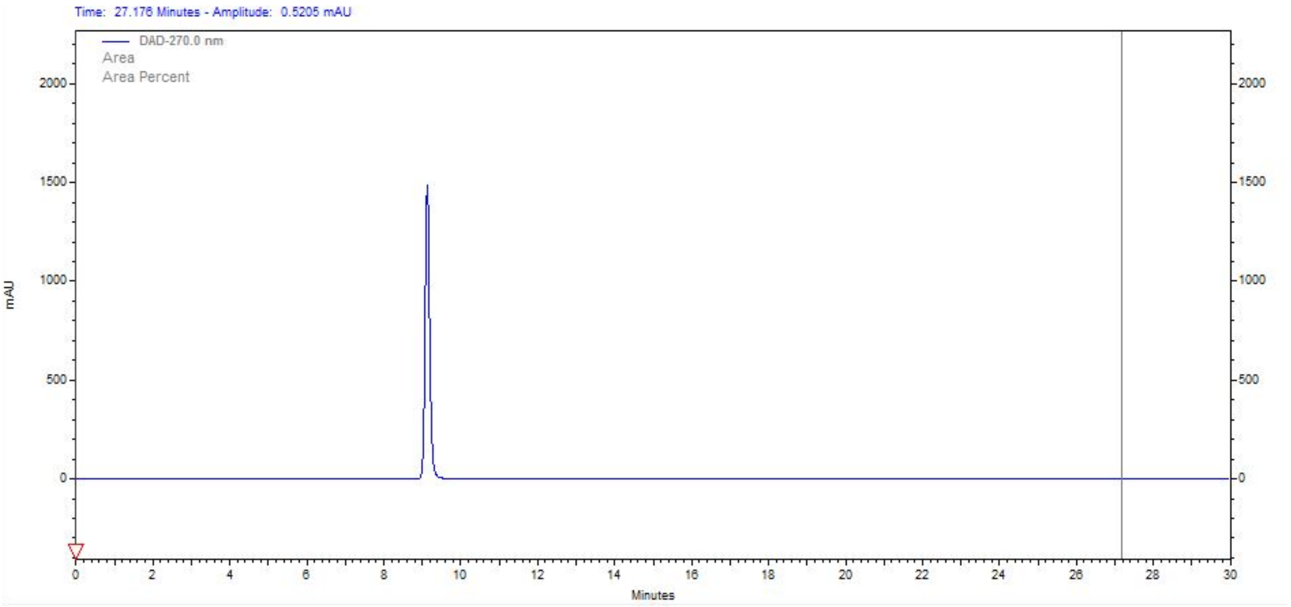

References

(1) Hananya, N.; Reid, J. P.; Green, O.; Sigman, M. S.; Shabat, D. Rapid Chemiexcitation of Phenoxy-Dioxetane Luminophores Yields Ultrasensitive Chemiluminescence Assays. *Chem Sci* **2019**, *10* (5), 1380–1385. https://doi.org/10.1039/c8sc04280b.
